# Chromatin occupancy and target genes of the haematopoietic master transcription factor MYB

**DOI:** 10.1038/s41598-021-88516-w

**Published:** 2021-04-26

**Authors:** Roza B. Lemma, Marit Ledsaak, Bettina M. Fuglerud, Geir Kjetil Sandve, Ragnhild Eskeland, Odd S. Gabrielsen

**Affiliations:** 1 Department of Biosciences, University of Oslo, Blindern, PO Box 1066, 0316 Oslo, Norway; 2 Centre for Molecular Medicine Norway (NCMM), Nordic EMBL Partnership, University of Oslo, 0318 Oslo, Norway; 3 Institute of Basic Medical Sciences, Department of Molecular Medicine, University of Oslo, Blindern, PO Box 1112, 0317 Oslo, Norway; 4 Terry Fox Laboratory, BC Cancer, Vancouver, BC V5Z 1L3 Canada; 5 Department of Medical Genetics, University of British Columbia, Vancouver, BC V6T 1Z4 Canada; 6 Department of Informatics, University of Oslo, Blindern, PO Box 1080, 0371 Oslo, Norway; 7 Centre for Cancer Cell Reprogramming, Institute of Clinical Medicine, Faculty of Medicine, University of Oslo, Oslo, Norway

**Keywords:** Genome informatics, Cancer genomics, Oncogenes, Epigenetics, Gene regulation, Transcriptomics, Epigenetics, Transcription, Transcriptomics

## Abstract

The transcription factor MYB is a master regulator in haematopoietic progenitor cells and a pioneer factor affecting differentiation and proliferation of these cells. Leukaemic transformation may be promoted by high MYB levels. Despite much accumulated molecular knowledge of MYB, we still lack a comprehensive understanding of its target genes and its chromatin action. In the present work, we performed a ChIP-seq analysis of MYB in K562 cells accompanied by detailed bioinformatics analyses. We found that MYB occupies both promoters and enhancers. Five clusters (C1–C5) were found when we classified MYB peaks according to epigenetic profiles. C1 was enriched for promoters and C2 dominated by enhancers. C2-linked genes were connected to hematopoietic specific functions and had GATA factor motifs as second in frequency. C1 had in addition to MYB-motifs a significant frequency of ETS-related motifs. Combining ChIP-seq data with RNA-seq data allowed us to identify direct MYB target genes. We also compared ChIP-seq data with digital genomic footprinting. MYB is occupying nearly a third of the super-enhancers in K562. Finally, we concluded that MYB cooperates with a subset of the other highly expressed TFs in this cell line, as expected for a master regulator.

## Introduction

The transcription factor c-Myb (approved human symbol MYB), encoded by the *MYB* proto-oncogene, is highly expressed in haematopoietic progenitor cells and plays a key role in regulating the expression of genes involved in differentiation and proliferation of myeloid and lymphoid progenitors^1–5^. Hence, MYB has been described as a master regulator^6–9^. Overexpression of MYB blocks erythroid and myeloid differentiation^10,11^, whereas mice with reduced levels of Myb have reduced levels of cells of lymphoid origin^12^. Leukaemic transformation may be promoted in cases where high MYB levels are maintained^1,13^, which is the case in many human lymphoid and myeloid acute leukaemias^14^, where MYB may act as a critical mediator of oncogene addiction^15^.

The MYB protein harbours three functional domains: an N-terminal DNA binding domain (DBD), a central transactivation domain (tAD), and a C-terminal regulatory domain (CRD)^16^. The acidic tAD contains a well characterized short linear motif (SLM) in the form of a LxxLL motif^17^, which is kept exposed by its acidic environment^18,19^. Through its tAD, MYB interacts with the KIX-domain of the histone acetyl transferases p300 and CBP^17–21^, an interaction which is required for the induction of acute myeloid leukemia (AML) and appears to be a promising therapeutic target for AML treatment^22^. MYB has also been shown to recruit the HAT CBP to the *TAL1* oncogene, resulting in extensive H3K27ac and the generation of an oncogenic super-enhancer^8^.

The DBD of MYB is highly conserved and consists of three tandem 50 amino acid repeats termed R1, R2, and R3, the first of which is deleted in the AMV and E26 oncoproteins^16,23^. Each of the three repeats adopts a helix-turn-helix-related secondary structure and the R2 and R3 repeats are required and sufficient for complex formation between MYB and DNA^20,28,29^. The MYB DBD shares sequence similarity with the SANT domain found in several chromatin-modifying enzymes and thought to bind to histone tails^24,25^. Two studies have shown that the MYB DBD, in addition to binding to DNA, also interacts with histone tails^26–28^. We recently showed that a mutant of MYB, D152V, causing haematopoietic defects in mice by an unknown mechanism^29^, had weakened interaction with histone H3 while its DNA binding was intact^30^. Based on the effect the mutant had on the MYB specific transcriptome and chromatin accessibility, we concluded that MYB acts as a pioneer factor and that D152V impairs this function specifically^30^.

Given all the critical MYB-dependent functions, it is important to know its chromatin occupancies because this is relevant for both the identification of its direct target genes as well as for judging its pioneer function. We have previously argued that a combination of chromatin immunoprecipitation (ChIP) and MYB knockdown may represent a powerful approach to identify a core collection of MYB target genes^31^. Here we analyse a new ChIP-seq dataset in combination with previous RNA-seq and ATAC-seq (Assay for Transposase Accessible Chromatin followed by high-throughput sequencing) datasets, allowing us to address several questions regarding the chromatin action of MYB.

## Results

### Determining the genomic profile of MYB

K562 is an interesting model cell line for studying human MYB. In addition to being an ENCODE (Encyclopedia of DNA Elements) model with extensively characterized epigenetic profiles^32,33^, K562 expresses high levels of MYB. According to Fantom5 (Functional Annotation of The Mammalian Genome), MYB is among the eight most highly expressed transcription factors (TFs) in K562 (https://fantom.gsc.riken.jp/5/sstar/FF:10824-111C5; https://fantom.gsc.riken.jp/5/sstar/FF:10826-111C7). We have previously generated both RNA-seq and ATAC-seq data from K562-derived cell lines subjected to MYB knockdown and rescue^30^. To extend and further explore these data sets, we needed a comparable ChIP-seq dataset for MYB.

**Figure 1:**
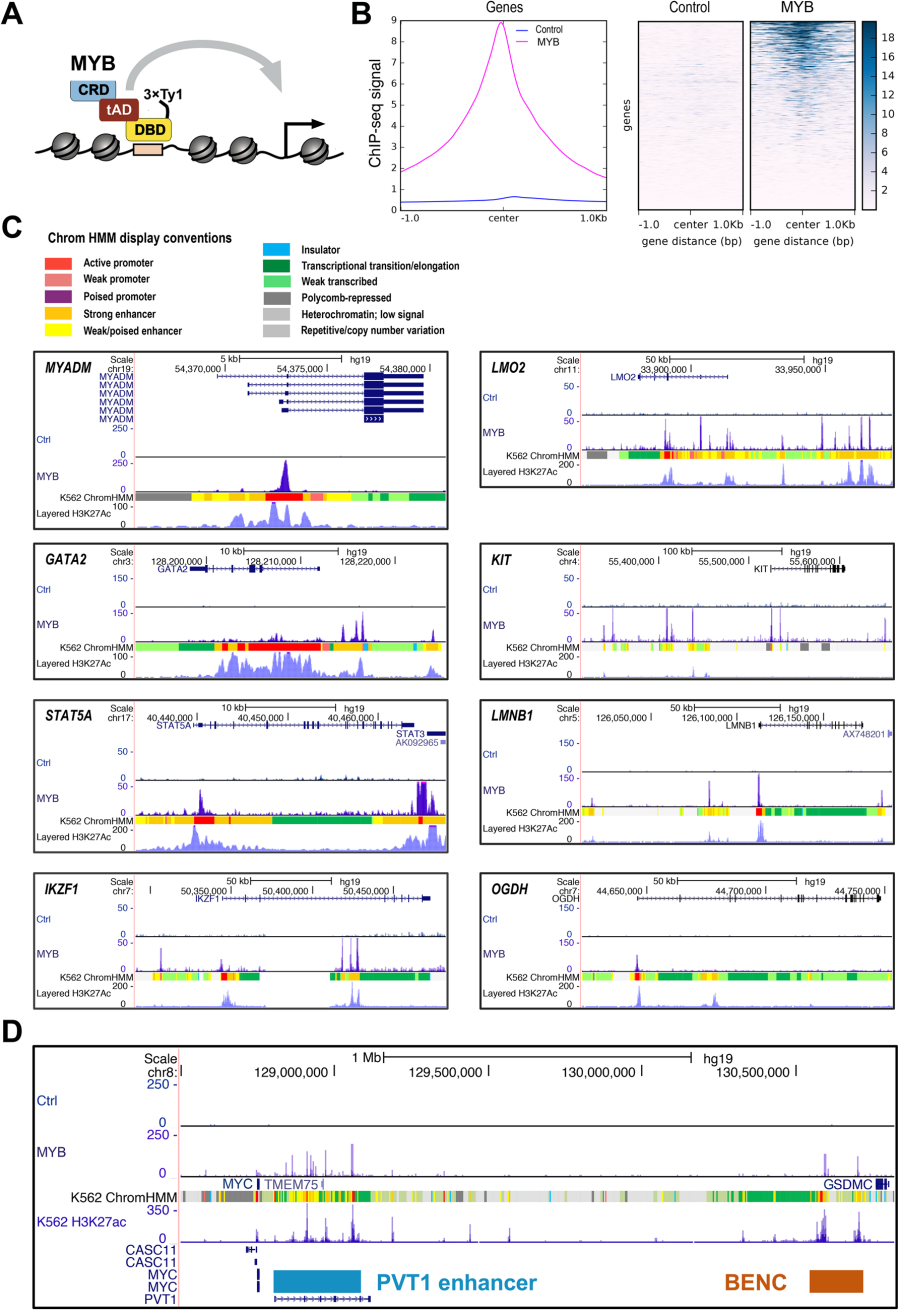
MYB occupies genomic loci of its target genes. (**A**) Model showing the full length MYB with N-terminal 3×Ty1-tag used to generate the current MYB ChIP-seq data from K562 cells. In the model the N-terminal DNA binding domain (DBD), the central transactivation domain (tAD) and the C-terminal regulatory domain (CRD) of MYB are shown. (**B**) An average ChIP-seq signal in K562 cells both from the control cell line and the stable cell line expressing N-terminally 3×Ty1-tagged full length MYB. Left panel shows a line plot showing the intensity of MYB ChIP-seq signals centred at the TSS of all genes. The heatmap shows average ChIP-seq signals at and ± 1 kb around the TSS of all genes. We used Ensembl human reference genome annotation (GRCh37 release 87) as regions for calculating the ChIP-seq signal enrichment at and ± 1 kb around the TSS. The line plot and heatmap were made using deepTools2 v3.3.0^77^. (**C**) UCSC tracks showing MYB occupancy at genomic loci of some of previously reported MYB target genes. MYB ChIP-seq peaks at the promoter regions of *STAT5A*, *MYADM* and *OGDH* and MYB ChIP-seq peaks both at the promoter and nearby or distal enhancer regions of *LMO2*, *KIT*, *LMNB1*, and *IKZF1* loci along with K562 chromHMM chromatin segmentation tracks are displayed. Color guide for display conventions used by chromHMM is provided. (**D**) MYB occupancy at *MYC* locus. Myb ChIP-seq peaks show occupancy at BENC and PVT1 enhancer regions located downstream of the *MYC* gene. Visualization of the tracks were made using the UCSC genome browser^76^ (https://genome.ucsc.edu/) by creating a UCSC session containing the K562 control and MYB ChIP-seq bigwig files from the current study.

Analysis of MYB occupancy in chromatin has been hampered by lack of ChIP-grade antibodies, although the Myb1.1 antibody has been used for local ChIP experiments^34,35^. This problem has been bypassed by performing ChIP against the estrogen receptor (ERα) in cells with a considerably truncated MYB fused to the ligand binding domain of ERα^36^. Recently, an antibody against MYB phosphorylated in Ser11 (Abcam [EP769Y] ab45150) has been successfully used in generating MYB ChIP profiles in some cell lines^8,37^. In order to determine the occupancy of full-length MYB independent of phosphorylation status, we decided to use the same K562 derived cell-line as for RNA-seq and ATAC-seq, where a 3×Ty1-tagged MYB (Fig. 1A) is expressed at a level close to endogenous MYB^30^. The Ty1-tag has been found to be a particularly suitable tag for ChIP^38^.

The individual replicates for the ChIP-seq data from K562 cells expressing Ty1-tagged MYB show high correlation with each other (Supplementary Fig. S1A left). The correlations between individual replicates of ChIP-seq data for the control cell line (K562 cells expressing the empty vector) were quite low reflecting the very low background (Supplementary Fig. S1A right).

### MYB occupies both promoter and enhancer regions in reported target genes

The line plots and heat maps (Fig. 1B, Supplementary Fig. S2A-B) show a clear enrichment of ChIP-seq peaks around the TSS of genes globally in K562 cells, whereas the control cell line with the empty vector (with 3×Ty1-tag) showed almost no enrichment, consistent with a very low background. This indicates that the current MYB ChIP-seq data is of high quality, where almost no regions are bound in the control data set.

The MYB peaks of the three replicates were compared using the intervene tool with a setting that required at least 50% physical overlap between peaks to count them as corresponding peaks. With this stringent approach, we obtained a total of 22,780 MYB ChIP-seq peaks that are shared by all three biological replicates (Supplementary Fig. S1B left). These regions were used for downstream analysis. As an extra quality control we included an additional ChIP-seq peak calling step with q-value cut-off < 0.005 and counting peaks that are consistent in all three biological replicates. Even with this extremely stringent cut-off, we found a large number of high quality MYB ChIP-seq peaks (a total of 17,172 MYB ChIP-seq peaks, Supplementary Fig. S2C), which when aggregated revealed a clear and centred peak for the MYB ChIP-seq data, whereas the control data showed no enrichment (Supplementary Fig. S2D).

As a first examination of the biological relevance of the MYB profiles, we examined the MYB occupancy around a selection of previously reported MYB target genes^7,30,31,39–41^. Here, we took advantage of publicly available chromHMM chromatin segmentation data for K562. The chromHMM algorithm defines different chromatin states by employing multivariate HMM (Hidden Markov Model) to associate combinations of chromatin modification patterns with specific chromatin states^42^. At many of these MYB target genes’ loci, MYB peaks were found close to the TSSs of the genes as well as at upstream enhancer regions as indicated by the chromHMM segmentation tracks (Fig. 1C). For example, MYB showed a strong peak close to the promoters of *MYADM* and *OGDH*, whereas in the case of *STAT5A, LMO2*, *KIT*, *LMNB1*, and *IKZF1*, MYB showed occupancy both at the promoter and at distal or nearby enhancer regions. In *GATA2* MYB peaks were found mainly at an enhancer region upstream of its TSS. Overall, we conclude that MYB showed relevant occupancies of loci that correspond to previously reported MYB target genes^7,30,31,39–41^. Some of these MYB target genes, namely *LMO2*, *KIT*, *STAT5A*, and *LMNB1,* belong to a subset of target genes that the “pioneer” mutant of MYB (D152V) is incapable of activating^30^. As validation, peaks from Fig. 1C were analysed and confirmed by an independent ChIP-qPCR experiment (Supplementary Fig. S3).

The *MYC* gene was clearly responsive to MYB knockdown and rescue^30^ and has also previously been reported to be a target gene of MYB^43,44^. A manual inspection of 100 kb around the *MYC* gene did reveal some MYB peaks, but they were not as striking as for many other target genes. Is *MYC* a target gene of MYB? The enhancer landscape of *MYC* in haematopoietic progenitor cells is complex, including a super-enhancer region, named as BENC (for **B**lood **EN**hancer **C**luster), located 1.7 Mb downstream of the gene^45^. The BENC region contains two strong MYB ChIP peaks (Fig. 1D). In addition, MYB also occupies another cluster of proximal enhancers (PVT1 enhancer region, Fig. 1D). It appears therefore that MYB does occupy key regulatory elements controlling *MYC* expression. This example illustrates how challenging it is to judge target genes when really long-range interactions are involved.

**Figure 2:**
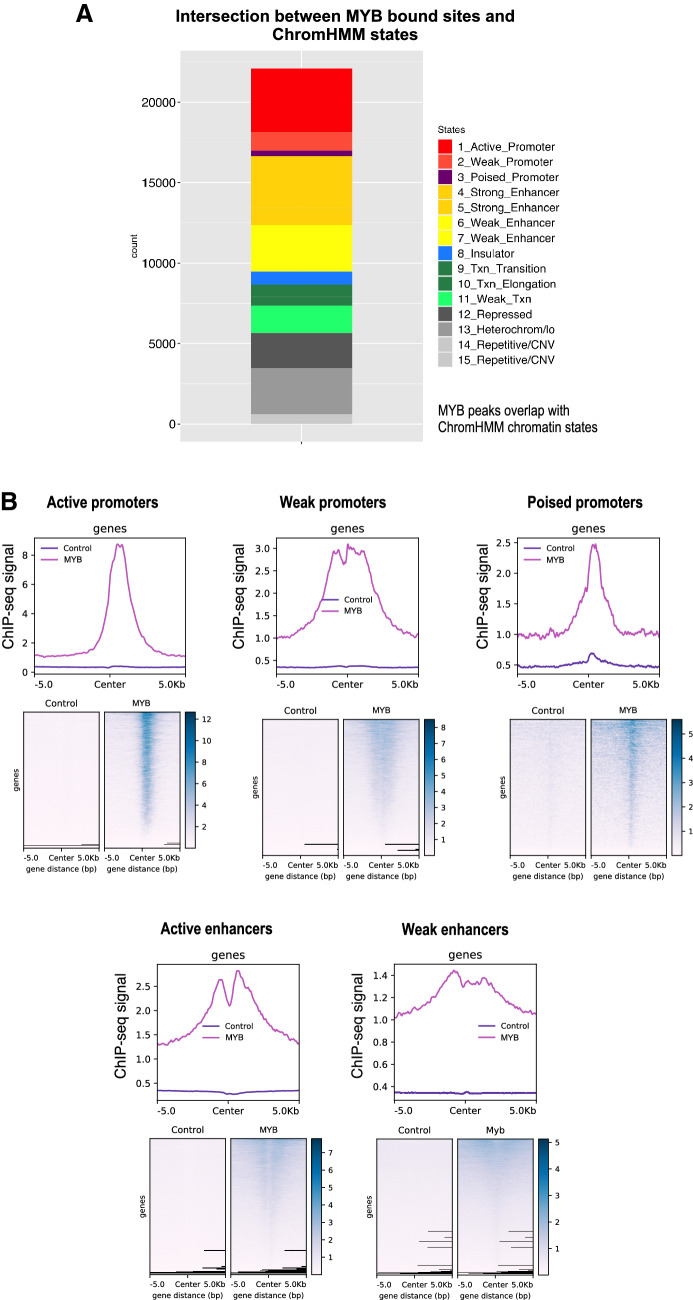
Distribution of MYB ChIP-seq peaks across chromHMM chromatin states in K562. (**A**) Distribution of overlaps between MYB ChIP-seq peaks and chromHMM chromatin states from K562 cells (UCSC Accession: wgEncodeEH000790), the stacked barplot was generated using the R environment v3.6.1 and ggplot2 package v3.3.2. To investigate overlap between the current MYB ChIP-seq peaks and chromHMM chromatin states from K562, we used bedtools v2.17.0^74^. (**B**) Heatmaps and line plots showing MYB ChIP-seq aggregated signals aligned at and ± 5 kb around the center of different chromatin state intervals from K562 chromHMM segmentations. Heatmaps and line plots were made using deepTools2 v3.3.0^77^.

Examination of the global distribution of MYB peaks (Fig. 2A) showed that a large fraction was enriched in promoters (24.7% including active, weak and poised promoters) and an even larger fraction in enhancer regions (32.5% including strong and weak/poised enhancers). We also noticed that a significant fraction of MYB were found at heterochromatin and repressed regions of chromatin (22.9%).

A more detailed set of enrichment profiles of MYB peaks relative to the control is illustrated in Fig. 2B, one for each of the subgroups of enhancers and promoters. Active promoters show the most focused profile and is stronger than the other promoter classes. Similarly, strong enhancers show higher MYB enrichments than weak enhancers.

### Classes of chromatin occupancy of MYB

**Figure 3:**
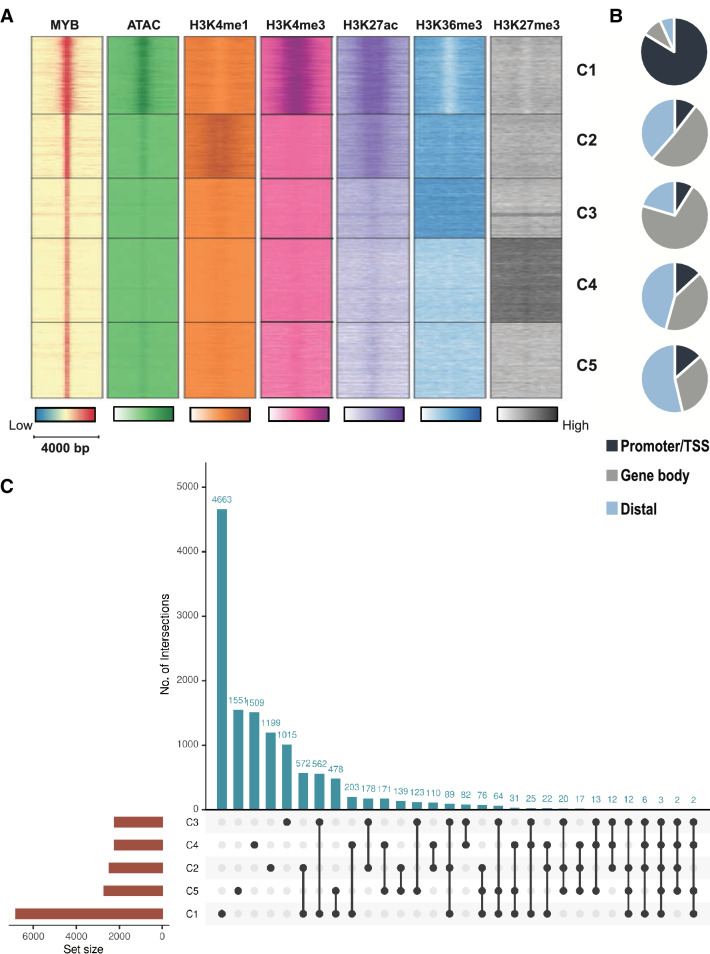
Epigenome-wide clustering of MYB. (**A**) Heatmap displaying the current MYB ChIP-seq signal, public ChIP-seq signals for H3K4me1 (GSM733692), H3K4me3 (GSM733680), H3K27ac (GSM733656), H3K36me3 (GSM733714), and H3K27me3 (GSM733658) ChIP-seq, and ATAC-seq signal from our previous data (GEO accession: GSE92871) within a 4 kb window around the summit of MYB bound regions. We identified five clusters as indicated on the heatmaps, generated by ChAsE 1.1.2^46^ (termed C1-C5 in the figure). (**B**) Distribution of MYB binding sites between TSS/promoter, gene body or distal regions in each cluster C1-C5. (**C**) An upset plot showing overlaps of the number of genes associated with each C-group. The upset plot was generated using the Intervene upset module v0.6.4^78^.

The global distribution of MYB led us to investigate in more detail the epigenetic profiles of MYB occupancies, more specifically in relation to public ChIP-seq datasets for H3K4me1, H3K4me3, H3K27ac, H3K36me3, and H3K27me3 in K562 cells. In addition, we included our previous ATAC-seq data. Using the ChAsE tool^46^, five clusters were identified, termed C1–C5 (Fig. 3, and Supplementary Fig. S4). C1 appears to be enriched for promoters due to its high level of H3K4me3 and strong ATAC signal. C2 appears to be dominated by active enhancers reflected in the high level of the enhancer-marks H3K4me1 and H3K27ac. C3 looks like an intragenic cluster since it is enriched for H3K36me3 over a broad area. C4 corresponds to repressed chromatin as judged by its broad enrichment of H3K27me3. Finally, the C5 cluster appears as unmarked chromatin. This interpretation is also supported by the pie-diagrams in Fig. 3B.

We used STITCHIT to identify genes associated with the MYB peaks (Fig. 3C). STITCHIT is an algorithm and a resource that employs epigenomic signals in combination with gene expression of a distinct gene to identify gene-specific regulatory elements (REMs)^47^. Among the 12 950 genes found, the largest group associated with the C1 promoter cluster. A large fraction of genes associated with the C2 enhancer cluster also had MYB peaks in the C1 cluster (572 of 2458). Figure 1 include several examples of genes with the latter type of occupancies.

Having genes associated with the C-groups, we asked whether this clustering reflect any functional features as revealed by a GO-term analysis. It should be noticed here that the presence of a MYB peak does not imply that the associated genes are always dependent on MYB for their expression. MYB may contribute to their expression to very different degrees and in some cases not at all, or only indirectly through its pioneer function. The GO-terms of each cluster revealed that the C2 enhancer cluster is enriched for functions linked to the development of hematopoietic cells, such as erythrocyte development and differentiation, T cell selection and activation and myeloid cell development (Supplementary Fig. S5A). The other groups showed enrichments that were less obviously related to MYB functions. While a GO-term analysis compares gene sets to unravel functional enrichments, other enrichment tools have been developed to compare genomic loci rather than genes. We used one of these, LOLAweb^48^, on the C-groups in order to compare our genomic MYB profiles with other epigenetic profiles in the public domain. The results suggested that the C2 group is very cell-type specific, showing closest similarity with other K562 datasets, many of which are TF ChIP-profiles (Supplementary Fig. S5B) and with a p300 ChIP-profile as the highest ranked similarity. The C1 group, in contrast, seems to overlap with active regions in multiple different cell types, where TSS-segments and H3K4me3 ChIP-profiles show closest similarity. Our interpretation is that MYB occupies many cell-type specific enhancers in K562 (the C2-group) as well as many possibly house-keeping promoters (the C1-group). The set of genes repressed (the C4 group) is also common to many cell-types, while the intragenic cluster (C3-group) and the C5 group (unmarked chromatin) are more cell-type specific.

### Enrichment of TF-binding sites in MYB-peaks—evidence for direct binding

**Figure 4:**
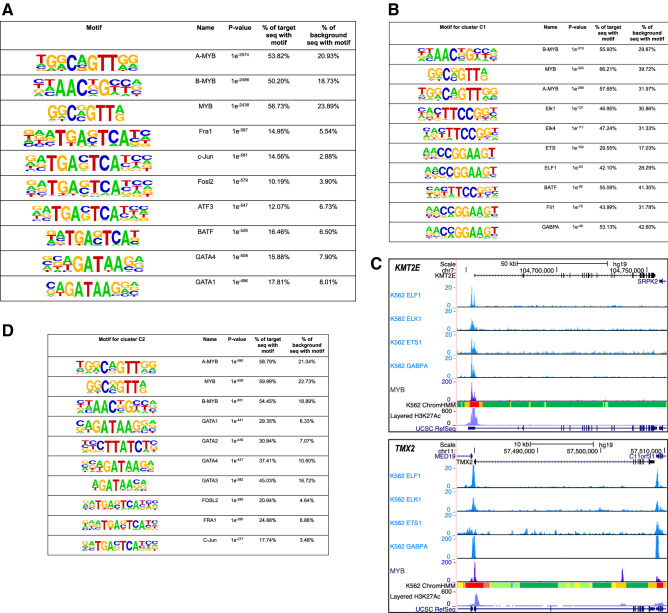
Identified MYB ChIP-seq peaks are evidence for its direct binding. (**A**) Enrichments of known motifs at MYB occupied regions from three biological replicates in K562 cells (n = 22,780). (**B**) Enrichments of known motifs at MYB occupied regions belonging to MYB cluster C1 from Fig. 3A. (**C**) Occupancy of MYB at *KMT2E* and *TMX2* loci. Publicly available ChIP-seq tracks for ETS-related factors from K562 cells are displayed along with the current MYB ChIP-seq data. Visualization of the tracks were made using the UCSC genome browser^76^ (https://genome.ucsc.edu/) by creating a UCSC session containing the K562 control and MYB ChIP-seq bigwig files from the current study along with the bigwig files of publicly available K562 ChIP-seq data for ELF1 (ENCFF921NSY), ELK1 (ENCFF390PFQ), ETS1 (ENCFF448HHF), and GABPA (ENCFF450AAJ). (**D**) Enrichments of known motifs at MYB occupied regions belonging to MYB cluster C2 from Fig. 3A. Enriched binding motifs for panel A, B and D are arranged in descending order based on their p-value. Motif analyses around MYB binding sites were made using the HOMER program version 4.9^82^.

The regions occupied by MYB showed high enrichments (50–57%) for motifs binding the MYB paralogs A-MYB (MYBL1), B-MYB (MYBL2) and MYB, all three having quite similar DNA-binding preferences^49^ (Fig. 4). This suggests that MYB ChIP-peaks are dominated by direct MYB binding to DNA. Although clearly behind these first three, other TF binding sites were also found enriched (Fig. 4A). Several quite similar motifs binding to members of the bZIP AP-1 group made up five of the top ten positions on the list (FRA1, JUN, FOSL2, ATF3, BATF), all with a core consensus sequence TGASTCA. Finally, motifs for the master regulators GATA1 and GATA4 also showed significant enrichment. These co-occurrences may reflect a high level of co-occupancy between these factors and MYB in K562 cells.

The same type of motif analysis in the C-groups identified in Fig. 3 showed for the C1 promoter group that MYB-binding motifs remained on the top (in 56–66% of the peaks) (Fig. 4B). In addition, a cluster of ETS-related motifs had quite high frequency, all with the common core sequence TTCCGG, representing putative members of this family (ELK1, ELK4, ETS, ELF1, BATF, FLI1, and GABPA). This may reflect a high level of co-occupancy of MYB and ETS-factors in the C1 promoter group. Two examples of co-occupancy using publicly available ChIP-seq datasets, at the *TMX2* and *KMT2E* genes, are shown in Fig. 4C. Both the *TMX2* and *KMT2E* genes are among the significantly down-regulated genes in response to endogenous MYB knockdown in K562 cells^30^.

A similar analysis of the C2 enhancer group showed the same top enrichment of MYB related motifs (54–60%), but here the group of GATA factors came second in frequency and the AP-1 type third in the ranking. The cooperation with GATA factors is addressed below. Similarly, in the C3 group the AP-1 group of motifs came second after the top MYB related motifs (Supplementary Fig. S6). The same was true for the C4 group.

### Colocalization with other TFs

**Figure 5:**
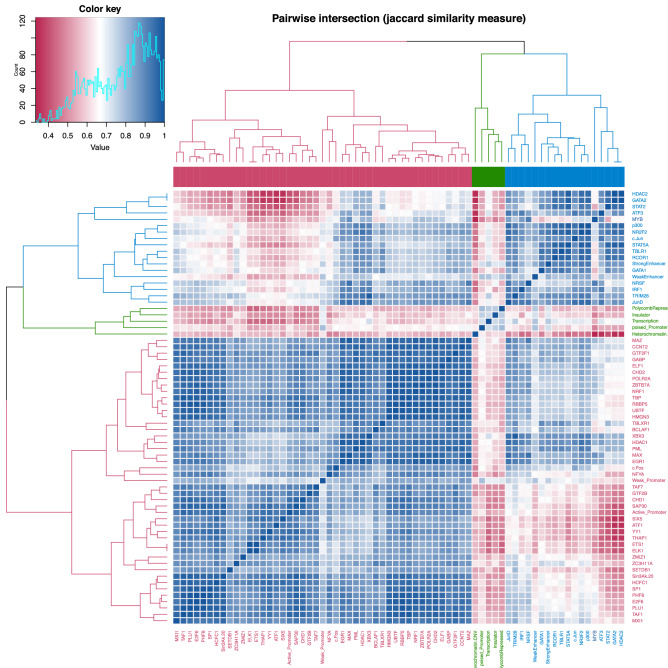
Hierarchical clustering of MYB ChIP-seq data with publicly available ChIP-seq data. Pairwise intersection profiles between MYB and that of publicly available ChIP-seq peaks in K562 cells with jaccard similarity measure was obtained and hierarchical clustering was employed using intervene v0.6.4^78^. The heatmap was generated from the resulting matrix using the intervene shinyApp (https://asntech.shinyapps.io/intervene/).

Motif enrichments identify potential cooperative TFs. To address if co-occupancy actually occurs at these genomic loci, we analysed the pairwise similarity between the MYB ChIP-seq profiles and those of other TFs and chromatin-associated proteins in K562. From the hierarchical clustering, we observed one big clade (red branch) and two smaller clades (green branch and blue branch), making up three individual clusters (Fig. 5). TFs in the red branch clustered with the chromHMM active and weak promoter genome segmentations, whereas TFs in the blue branch clustered with the chromHMM strong and weak enhancer genome segmentations. The latter included TFs acting as master regulators of haematopoiesis (such as GATA1 and GATA2), as well as the enhancer-enriched histone acetyltransferase p300^50^. We refer to the blue clade as an “enhancer cluster” and the red clade as a “promoter cluster”, the latter because it contains several factors associated with TSS regions (POLR2A, TBP, TAF7, GTF2B, GTF2F1, and CHD1). The MYB ChIP-seq peaks clustered with the “enhancer cluster”. In contrast to the C-clusters above, this clustering is based on colocalization with other TFs, not allowing peaks to belong to multiple clusters. This puts MYB in a cluster enriched for enhancer regions, in line with the C2 group above. It is also consistent with the motif analysis where the second ranked enriched motif in the C2 group was for GATA-family factors. The ETS-family factors (ELK1, ETS1 and ELF1) with motifs enriched in the C1 group are found in the red clade (“promoter cluster”).

**Figure 6:**
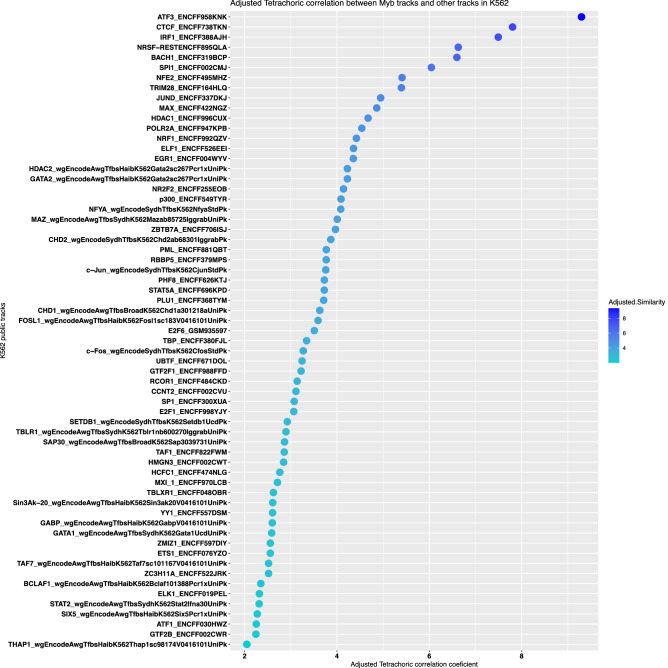
Co-localization of MYB with other TFs, co-factors and proteins of genomic relevance. Co-localization analysis of MYB ChIP-seq peaks with publicly available ChIP-seq data from K562 was made using the GSuite HyperBrowser (https://hyperbrowser.uio.no/). The tetrachoric correlation was used as a pairwise similarity measures between genomic intervals from MYB and that of the publicly available targets for K562 (Supplementary Table S1). The resulting correlation table was used to generate the dotplots using the R environment v3.6.1 and ggplot2 package v3.3.2.

A slightly different approach to address the colocalization issue is shown in Fig. 6. Here, we collected a set of publicly available ChIP-seq datasets for 63 targets (TFs, co-factors and proteins of genomic relevance) in addition to our current MYB ChIP-seq data, and carried out genomic colocalization analysis using the GSuite HyperBrowser^51^, a web tool that allows the interrogation of TF cooperativity at the genomic co-occupancy of these TFs. For this, we used the tetrachoric similarity measure implemented in the GSuite HyperBrowser. Several factors showing most frequent colocalization with MYB are either in the top 10 or top 20 of the HOMER known motifs (Fig. 4A) or motifs from the C groups (Fig. 4B,C and Supplementary Fig. S6), indicating that the various colocalization and similarity comparisons are in agreement. One example is ATF3 with an AP1-type binding motif that clustered in the blue ‘enhancer clade’ together with MYB (Fig. 5) and was ranked here at the top of factors that colocalize with MYB (Fig. 6). More top ranked examples include IRF1, NRSF, TRIM28, and JUND (Fig. 6), which were all consistently found in the same ‘enhancer clade’ clustering with MYB (Fig. 5). Factors from the red ‘promoter clade’ in Fig. 5, such as MAX, HDAC1, POLR2A, NRF1, ELF1, and EGR2 are likewise in the top 15 factors in Fig. 6. This entails that these factors tend to colocalize with MYB more frequently than others.

We analysed further the genomic regions where MYB colocalized with each of the top five TFs in Fig. 6, i.e. ATF3, CTCF, IRF1, NRSF, and BACH1. We associated these shared coordinates with target genes using STITCHIT and carried out GO enrichment analysis. Regions with co-localized MYB and ATF3 and with co-localized MYB and CTCF are both dominated by enrichments for chromatin organization and expression, epigenetic and/or transcriptional regulations, in line with MYB being a master regulator (Supplementary Fig. S7). A few illustrating examples are shown in Supplementary Fig. S11. Regions co-occupied by MYB and BACH1 show top enrichment of GO-terms for transcriptional regulation (Supplementary Fig. S7), while in regions co-occupied by MYB and IRF1 immune related signalling terms dominate. Finally, regions co-occupied by MYB and NRSF are enriched for GO-terms associated with macromolecule/protein modifications (Supplementary Fig. S7).

### Are MYB target genes direct targets?

Based on the combination of RNA-seq data, with knockdown and rescue of MYB^30^, and the MYB ChIP data, we were able to ask several additional questions. The first obvious question was: How many of the MYB target genes defined from our previous RNA-seq data set are direct target genes? A gene whose expression is altered after knockdown of MYB for 24 h, could show this response due to a direct or indirect effect of reduced MYB levels. We have previously argued that a combination of ChIP and knockdown allows identification of direct MYB target genes^31^. While the latter provide strong evidence for a functional link, the first provide evidence for a direct physical link.

**Figure 7:**
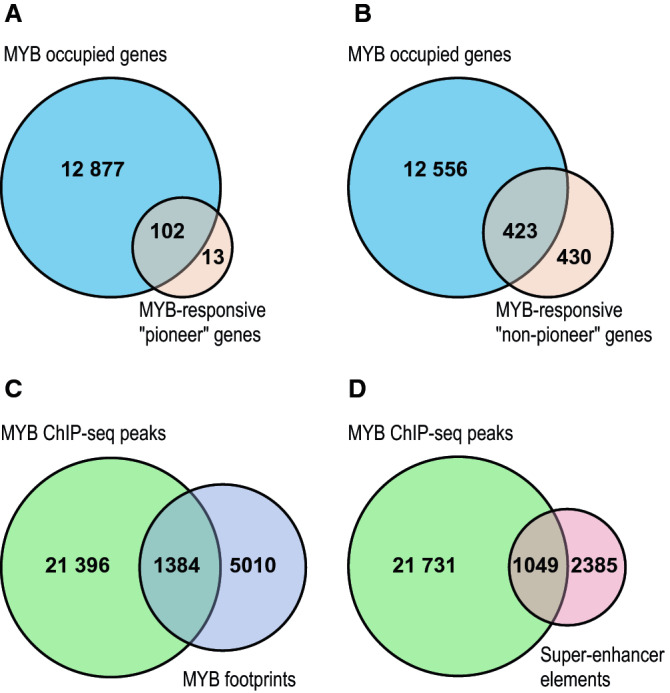
Proportion of direct and indirect MYB target genes. (**A**) Intersection between MYB occupied genes and MYB’s pioneer target genes. (**B**) Intersection between MYB occupied genes and MYB’s non-pioneer target genes. The pioneer and non-pioneer target genes were identified from our previous RNA-seq data in K562 (GEO accession: GSE85187), where an endogenous MYB KD affected genes were rescued by a WT MYB but not a pioneering function deficient D152V mutant MYB, hence corresponding to MYB responsive pioneer target genes. In contrary, the non-pioneer target genes correspond to those target genes whose MYB KD related phenotype was rescued by both WT and D152V MYB. (**C**) Intersection between MYB ChIP-seq peaks and MYB’s digital footprints in K562. Computationally predicted digital footprints of MYB previously reported in our lab^39^ was used to investigate genomic overlap with the current MYB ChIP-seq peaks. (**D**) Intersection between MYB ChIP-seq peaks and MYB super-enhancer elements in K562. Super-enhancer elements in K562 reported in Qian et al. was used to investigate the overlap with the current MYB ChIP-seq peaks^58^. The genomic Overlaps between MYB occupied regions and that of MYB DGF or K562 SEs were investigated using bedtools v2.17.0^74^. Example UCSC tracks illustrating each of the overlapping and non-overlapping condition for panel (A-D) are provided in Supplementary Figure S10.

We examined first the list of target genes dependent on the pioneer function of MYB, i.e. their expression is not rescued after knockdown by a D152V mutant version of MYB. This group of target genes is assumed to be particularly important for the ability of MYB to regulate cell differentiation and hematopoietic development^30^. Being dependent on a pioneer function of MYB, we have dubbed them “pioneer” genes (Supplementary Fig. S8). We used STITCHIT annotated MYB ChIP-seq peaks (to define the subgroup of peaks associated with a gene) and investigated their overlap with the list of “pioneer” target genes. 102 out of 115 “pioneer” genes had regions occupied by MYB (Fig. 7A). We conclude that a majority of the “pioneer” genes are true target genes according to the proposed criteria.

In the larger list of putative target genes of MYB^30^, which were rescued by both a wild-type and a D152V mutant of MYB (“non-pioneer” genes), the picture was different, with only about 50% of the putative target genes meeting the criteria for a direct target gene (Fig. 7B). This suggests that in the larger list of genes affected by MYB knockdown, we find as many indirect as direct target genes of MYB.

### How precise is digital genomic footprinting?

We have previously exploited digital genomic footprinting (DGF) analysis to obtain a global picture of MYB genome occupancy in several hematopoietic cell lines, including K562 where we identified 6394 conserved footprints sharing a MYB signature^39^. This is less than a third of the MYB ChIP-seq peaks in this work (n = 22,780). DGF has been a highly promising procedure for mapping TFs in the genome^52–54^, but also a controversial method^55^. With our MYB ChIP-seq dataset, we could directly compare the results of the two methods (Fig. 7C). Among the DGF footprints (n = 6394), 22% (n = 1384) were confirmed by the present ChIP-seq data. This overlap may still be sufficient to predict a reasonable fraction of the genomic landscape of MYB. More problematic is the high frequency of predicted MYB footprints not confirmed by MYB ChIP (n = 5010). We have in this work used a quite stringent approach counting only MYB ChIP-seq peaks that are common between three biological replicates. The overlap between the DGF peaks and at least one of the ChIP replicates was 74%, but this improvement would be at the expense of poor reproducibility. One reason for the low number of DGF footprints may be because there we applied a conservation requirement severely reducing the number of DGF peaks^39^.

### Does MYB occupy super-enhancers?

Related to the concept of master regulators is the concept of super-enhancers (SEs), defined as large clusters of enhancers working together to efficiently drive the expression of genes with a key role in defining cell identity^56,57^. A direct role for MYB in the establishment of oncogenic SEs has been reported^8^. On this background we asked how frequently MYB is found associated with SEs in K562 cells, defined as in Qian et al.^58^. Using bedtools intersect, we observed 1049 overlapping regions between MYB ChIP-seq peaks (n = 22 780) and that of the SE elements in K562 (n = 3434) (Fig. 7D and Supplementary Fig. S9). This means that MYB is occupying nearly a third (31%) of the SEs in K562, consistent with its role as a master regulator.

### The role of MYB as a master regulator—other TFs as targets of MYB

**Figure 8:**
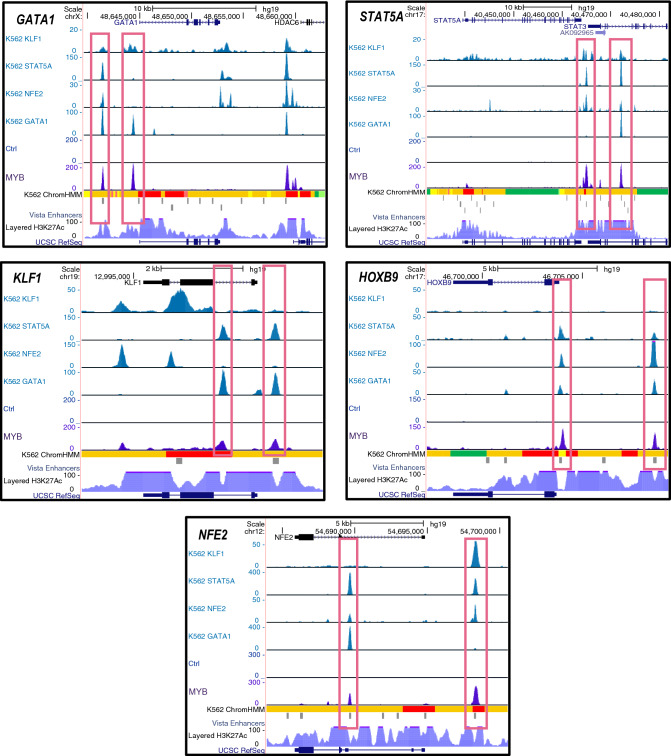
MYB has a master regulatory property. MYB occupies the promoter and enhancer regions of key regulators of gene programs in K562: *GATA1*, *STAT5A*, *KLF1*, *HOXB9*, and *NFE2* loci. Publicly available K562 ChIP-seq data for GATA1, NFE2, STAT5A and KLF1 are displayed along with the current MYB ChIP-seq data and K562 chromHMM chromatin segmentation tracks, the latter used to define enhancer regions. Visualization of the tracks were made using the UCSC genome browser^76^ (https://genome.ucsc.edu/) by creating a UCSC session containing the K562 control and MYB ChIP-seq bigwig files from the current study along with the bigwig files of publicly available K562 ChIP-seq data for KLF1 (ENCFF153SCC), STAT5A (ENCFF746KSV), NFE2 (ENCFF399JNI), and GATA1 (ENCFF724KSO).

The final question we asked was whether MYB clusters with other master TFs in K562? Our current understanding of cell type determination is that a small number of key TFs, typically master TFs, dominate in the control of the underlying gene expression programs^59,60^. These clusters of TFs will be expressed at relatively high levels and they will co-occupy many enhancers driving active genes. In addition, they also tend to regulate each other supporting their high expression levels. According to Fantom5, the top eight TFs expressed in K562 cells are GATA1, STAT5A, CTCFL (Boris), NFE2, PITX1, HOXB9, MYB, and KLF1 (https://fantom.gsc.riken.jp/5/sstar/FF:10824-111C5; https://fantom.gsc.riken.jp/5/sstar/FF:10826-111C7). Since MYB is among these top eight factors, we asked whether MYB also occupies the promoters or enhancers of some of the other dominating factors. This is indeed the case for several of them, such as GATA1, STAT5A, NFE2, HOXB9, KLF1, while others had no or quite weak MYB peaks (CTCFL (Boris) and PITX1) (Fig. 8). We also included public ChIP data available for GATA1, STAT5A, NFE2, and KLF1 in Fig. 8. Clearly, several of the enhancers upstream of the genes encoding these TFs are occupied by a subset of the highly expressed TFs, as expected. The upstream region of the *GATA1* gene, for example, shows two regions where one is occupied by KLF1, STAT5A, NFE2, GATA1, and MYB, while the other is occupied by KLF1, GATA1 and MYB (Fig. 8). The *NFE2* gene shows a similar pattern. This supports the notion that MYB operates as a master regulator in K562 cells and cooperates with a subset of the other highly expressed TFs in this cell line, presumably contributing to the determination of its cell type characteristics.

## Discussion

We have in this work addressed the chromatin occupancy of the transcription factor MYB in the model cell line K562 to better understand its function. Although MYB has been studied for decades since the discovery of the oncogenic chicken v-Myb, our understanding of its molecular function is still limited. In particular, our understanding of its chromatin action has been hampered by lack of ChIP-grade antibodies. However, we know that MYB may severely influence chromatin states. A noteworthy example is the reported role for MYB in super-enhancer initiation^8^. Here, acquired somatic mutations were shown to create binding sites for MYB leading to the formation of a super-enhancer upstream of the *TAL1* oncogene. Our previous finding that MYB acts as a pioneer factor^30^, also points to a role in modulating chromatin states. In the present work, we identified 22 780 bona fide MYB occupied sites in K562 cells and performed several bioinformatics analyses of its pattern of occupancy.

The epigenetic signature of the regions in which MYB peaks were found allowed us to classify its occupancies into five groups. The C1 promoter group was not only enriched for MYB-binding sites, but also for ETS-factor responsive elements. It is noteworthy that in one of the first discovered oncogenic MYB-containing viruses, E26, the v-Myb oncogene encodes a fusion between MYB and ETS1^61^. Therefore, the E26 may encode a protein representing an enforced interaction through fusion of two TFs that also cooperate under normal conditions in a more fine-tuned manner.

The C2 group of MYB peaks had features indicating enhancer function. GO-terms suggested that particularly genes associated with the C2 group had cell type specific functions linked to haematopoiesis, genes that would be expected to be under enhancer control. Noteworthy is also that the C2 group, in addition to being enriched for Myb-responsive elements, also showed enrichment of GATA-factor motifs. GATA1 is considered as the master TF in erythropoiesis and the highest expressed TF in K562^62,63^. Therefore, MYB may be cooperating with GATA1 in controlling key target genes specific for haematopoietic development. Noteworthy, a transforming *MYB-GATA1* fusion gene has been identified, here in cases of acute basophilic leukemia^64^. Since super-enhancers have a key role in defining cell identity^65^, we analysed MYB occupancy at SEs. About 30% of SEs were occupied by MYB, again supporting a key regulatory role.

According to our current understanding, cell identities are established by a set of highly expressed, SE-driven TFs that form interconnected gene-regulatory networks^59,60^. By enhancing the expression of each other, a robust network is maintained. Among the eight highest expressed TFs in K562, which included MYB itself, we found that MYB occupied promoters and enhancers of five of them (Fig. 8), supporting a role of MYB in maintaining K562 cell identity. Evidence for this role of MYB was recently reported in KBM7 cells, a near-haploid human cell line, which like K562 is a chronic myelogenous leukemia cell line^66^. Upon ablation of the MED14 subunit of the Mediator in KBM7 cells, a rapid collapse in the transcription of the *MYC* and *MYB* genes was observed, supporting their importance for defining cell identity.

A master regulator would be expected to show two features, (1) pioneer function and (2) target genes that are associated with rewiring of gene programs. Pioneer TFs have the intrinsic ability to access closed chromatin that are inaccessible to other TFs^67^. These factors thereby play a key role in rewiring of gene networks during cellular differentiation. Through the study of a specific point mutant of MYB, D152V, which we found interfered with the ability of MYB to bind to histones, we recently reported that MYB acts as a pioneer factor in K562 cells^30^. When we looked for possible rewiring functions in the group of direct “pioneer” genes activated by MYB, we found several TFs, chromatin modifiers as well as signalling proteins. The list includes DNA-binding TFs (MYC, STAT5A, PGR, GATAD2A, NFIA, and SNAI1), and components of transcriptional complexes, both specific ones (LMO2 and MAML1) and more general ones (TAF4, MED13L, CDK12, and SCAF4). A few epigenetic regulators are also included (KDM1B, MBTD1, and SMARCC1). Finally, several proteins involved in signalling pathways are present (KIT, MAP4K4, PAK2, CDC27, GRB10, ANKRD27, RAB5A, and SKP2).

A classical question is whether target genes are direct or indirect. While it is obvious that RNA-seq analysis after knockdown could identify both types, it has also become clear that not all TF binding events are necessarily functional in the sense of changing the expression of associated genes. Often a relatively small overlap has been observed between TF occupancy and the expression of neighbouring genes, in the order of 10–25% in higher eukaryotes^59^. One example is the mammalian master regulator MYOD1 (myoblast determination protein 1) where only ~ 4% of genes with a MYOD1 ChIP-peak in their vicinity showed significant changes in their expression after removal of MYOD1^68^. For MYB, we see a similar phenomenon since MYB was found associated with more than 12 000 genes, of which only 4% was found to significantly change expression upon MYB knockdown (Fig. 7). Still, the question of functionality may be more complex for a pioneer factor since a binding event may also cause changes in chromatin states that indirectly affect the subsequent expression patterns in a differentiation process. From our estimate of direct target genes, we concluded that the majority (89%) of the “pioneer” genes were direct targets of MYB, while only about 50% of the “non-pioneer” genes seem to be direct target genes (Fig. 7). While the “pioneer” group was dominated by MYB activated targets (76 of 102), the “non-pioneer” group showed the opposite trend, with a slight bias towards repressed genes.

We finally compared the present ChIP-seq data with that of a previous prediction of MYB occupancy based on Digital Genomic Footprinting (DGF). DGF has been somewhat controversial, praised by some and criticized by others^55^. It was recently the basis of an extensive analysis as part of the ENCODE phase 3 project^69^. One critical feature appears to be the residence time of the TF determining the time the factor occupies and protects its target sequence. We argued that the DNA binding properties of MYB might be favorable, since MYB (at least in vitro) binds to DNA in a two-step process, where a rapid formation of an unstable complex is followed by a slower transition to a stable complex^70^. The latter would be expected to give robust footprints. Still, the comparison shows only a moderate overlap between the core ChIP-peaks and the conserved DGF-peaks. We therefore argue that DGF cannot fully replace real ChIP-seq data.

In conclusion, our analysis of the chromatin occupancy landscape of MYB is supporting a key role of MYB as a master regulator that together with other highly expressed TFs in K562 contributes to defining the identity of these cells.

## Methods

### Cell culture and stable cell line generation

In this study, we used K562 (ATCC CCL-243 Homo sapiens bone marrow, chronic myelogenous leukaemia) cell line. The cell line was maintained in IMDM medium (Gibco #21056-023) containing 10% FBS (fetal bovine serum) (Saveen Werner, FBS-11A, lot CP17-1844) and 1% penicillin/streptomycin (Gibco # 15,140–122). K562 cells stably expressing an N-terminal Ty1-tagged human MYB (pEF1neo-3×Ty1-hcM) and an empty vector (pEF1neo-3×Ty1) were generated as described^30^.

### Antibody

Ty1 antibody was purified in house from Ty1 hybridoma (BB2) supernatant by murine LC-kappa CaptureSelect antibody affinity resin (ThermoScientific) according to manufacturer’s instructions. Peak fractions were pooled, concentrated on an Amicon Ultra-15 3 K and dialysed against 1× PBS and stored in frozen aliquots of 1 mg/ml.

### ChIP sample preparation and sequencing

Chromatin was prepared from K562 cells stably expressing pEFneo-3×Ty1-MYB and control pooled cell lines with stable expression of pEF1neo-3×Ty1. 2 × 10^8^ cells from each cell line were crosslinked with 1% formaldehyde (Thermo Fisher Scientific #28906) in PBS containing 50 mM HEPES pH 8 for 15 min. The crosslinking reaction was stopped by adding 0.125 M glycine and incubating for 5 min on ice. The cells were washed once with PBS and incubated on ice for 10 min in swelling buffer (25 mM HEPES pH 7.9, 1.5 mM MgCl_2_, 10 mM KCl, 0.1% NP-40, 0.2 mM PMSF, 1 mM DTT) supplemented with 1 × cOmplete EDTA-free Protease Inhibitor Cocktail (Roche #04693132001). Nuclei from each sample were isolated by douncing (25 strokes) with a tight pestle followed by centrifugation at 2500 rpm and 4 °C for 4 min. The nuclei were re-suspended in 3 ml sonication buffer (50 mM HEPES pH7.9, 140 mM NaCl, 1 mM EDTA, 1% Triton X-100, 0.25% Na-deoxycholate, 0.2% SDS, 0.2 mM PMSF, 1 mM DTT, and 1 × cOmplete EDTA-free Protease Inhibitor Cocktail). Chromatin concentration was measured at A_260_ using 5 µl isolated nuclei diluted to 1:100 in urea buffer (2 M NaCl and 5 M Urea). The chromatin concentration was adjusted to 2 µg/µl in sonication buffer followed by chromatin shearing by sonication with maximum power with 30 s ON and 30 s OFF at 4 °C for 15 cycles using Bioruptor Pico sonication system. After sonication, the samples were centrifugated at 16,000×*g* for 15 min at 4 °C. Fragment size of the sheared DNA was determined with 15 µl sheared DNA that was de-crosslinked with 200 mM NaCl overnight and digested with RNase A (Sigma # R6513) and Proteinase K (Sigma # P2308) before the DNA fragments were separated on a 1% agarose gel.

6000 µg sheared chromatin was immunoprecipitated with 30 µg Ty1 antibody conjugated to 200 µl protein G Dynabeads (Invitrogen #10004D). 300 µg BSA was added to block the beads. The SDS in the immunoprecipitation reaction was diluted to 0.04% by adding sonication buffer without SDS to a final volume of 15 ml. After immunoprecipitation, the samples were washed (1) twice with sonication wash buffer (50 mM HEPES pH 7.9, 140 mM NaCl, 1 mM EDTA, 1% Triton X-100, 0.1% Na-deoxycholate, 0.1% SDS), (2) once with wash buffer A (50 mM HEPES pH 7.9, 500 mM NaCl, 1 mM EDTA, 1% Triton X-100, 0.1% Na-deoxycholate, 0.1% SDS), (3) once with wash buffer B (20 mM Tris pH 8.0, 1 mM EDTA, 250 mM LiCl, 1% NP-40, 0.1% Na-deoxycholate), and (4) once with TE buffer (1 mM EDTA, 10 mM Tris pH 8.0). All washing steps except the last one were performed at 4 °C by incubating samples on a rotator for 10 min. The last wash was performed at room temperature without incubation, samples were then centrifuged at 1000 rpm for 2 min.

Immunoprecipitated DNA was eluted from beads in two fractions using 250 µl fresh elution buffer (0.1 M NaHCO_3_, 1% SDS) per fraction with brief application of vortex and incubation at room temperature with rotation for 15 min. Pooled eluted fractions and 1% input samples diluted to 500 µl with an elution buffer were de-crosslinked with 200 mM NaCl overnight. The next day, samples were digested with 100 µg RNase A for 0.5 h and another round of digestion in the presence of 200 µg Proteinase K for 1 h at 37 °C. DNA purification was performed using ‘ChIP DNA clean and concentrator KIT’ (Zymo research #D5205), according to the manufacturer’s protocol. The DNA was collected in low binding tubes by eluting with 15 µl low TE buffer ( 10 mM Tris–HCl pH 8.5, 0.1 mM EDTA) and stored at − 80 °C. The concentration of purified DNA was assessed using 1 ul of the eluted DNA with ‘Qubit dsDNA HS assay kit’ (Thermo Fisher Scientific #Q32851), following the manufacturer’s protocol. Sequencing libraries were prepared using the ThruPLEX low-input sample prep method at the Norwegian sequencing centre. A 75 bp single-end read ChIP-Seq data for the cell lines with three biological replicates were generated using Illumina NextSeq 500 (High output reagents) sequencer.

### ChIP-Seq analysis

Quality check and preprocessing of the raw sequencing reads, alignment to hg19 human reference genome, quality check of mapping and downstream processing of aligned reads was performed as described in^71^.

Peaks from individual replicates and pooled replicates were generated using MACS2 (version 2.1.0)^72^, narrow peaks were called using the parameters “-g hs -m 5 50 --bw 150 --fix-bimodal --extsize 100 --call-summits -q 0.01”. The resulting peaks were refined using the “Poisson Pvalue” (ppois) method under the bdgcmp function of MACS2. Refined peaks in bedGraph format were then converted to bigWig format using bedGraphToBigWig (version 4)^73^. Narrow peaks in bed format was subjected to bedtools merge function (version 2.17.0) to collapse intervals that overlap with each other^74^.

All the above analysis was performed in an automated workflow using snakemake version (version 5.5.4)^75^. Generated peaks from pooled replicates were visualized using the UCSC genome browser^76^.

### Correlation of ChIP-seq data between replicates

We used the plotCorrelation program under deepTools2 (version 3.3.0) to investigate correlation scores of genomic regions within each biological replicates^77^. The intervene venn module (version 0.6.4) with the parameters ‘--type bed --bedtools-options f = 0.5,r’ was applied to obtain at least 50% physical and reciprocal overlaps between the MYB peaks replicates as well as the control ChIP-seq peaks replicates^78^.

### Investigation of ChIP-seq profiles on genomic regions

ChromHMM chromatin segmentation data for K562 was obtained from the ENCODE project and the segmentation data was split into separate bed files corresponding to the different chromatin states. Average ChIP-Seq signal across MYB ChIP-seq intervals and/or ChIP-seq interval from control data were calculated using the computeMatrix tool from deepTools2 program by centering the ChIP-seq signals to (1) nearest TSS of all genes and (2) K562 chromHMM defined chromatin states. The corresponding signal matrices were visualized using the plotHeatmap and plotProfile tools under deepTools2 program^77^.

Coordinates for chromatin regions defined as super-enhancers (SEs) in K562 cells were obtained from Qian et al.^58^. Bedtools intersect was used to investigate the overlap between the K562 SEs and current MYB ChIP-seq peaks in K562 (n = 22,780). Similarly we investigated the overlap between MYB ChIP-seq peaks and digital genomic footprints predicted in K562 cells from Bengtsen et al.^39^.

### Genomic region annotations

In order to associate genomic coordinates corresponding to MYB ChIP-seq peaks, we used annotation coordinates from STITCHIT, a method that assigns genome-wide regulatory elements (REMs) to their target genes using paired expression data and epigenomics data deposited by ENCODE, Roadmap and Blueprint projects^47^. By taking a unified list of STITCHIT assigned REMs from these three projects using bedops (version 2.4.14)^79^, we annotated genomic regions from the MYB ChIP-seq data with the unified STITCHIT generated REMs using bedtools intersect.

### Myb peaks clustering with epigenomic data

We used ChAsE version 1.1.2^46^ for k-means clustering (k = 5) and to generate heatmaps of MYB ChIP-seq peaks, ATAC-seq data (GEO accession: GSE92871) from^30^, and K562 histone modification ChIP-seq data from the ENCODE project (GEO accession: GSM733692 (H3K4me1), GSM733680 (H3K4me3), GSM733656 (H3K27ac), GSM733714 (H3K36me3) and GSM733658 (H3K27me3)). Distribution of MYB peaks in each cluster C1-C5 between TSS/promoter regions (here delimited from 5000 bp upstream to 500 bp downstream of the TSS), within gene bodies, or distal regions was determined using bedtools intersect intervals^74^. STITCHIT annotations corresponding to MYB occupied regions in C1-C5 clusters were used and list of genes to investigate GO enrichments using PANTHER v11^80^. Overlap of list of genes between STITCHIT annotated C-groups was investigated using the intervene upset module^78^. Average profile plots of histone modifications and MYB signal were generated using SitePro in the Galaxy/Cistrome toolbox^81^. Enrichment of regions and cell type for cluster C1-C5 was investigated using the LOLAweb tool using ‘activeDHS_universe.bed’ provided by LOLAweb as a ‘background universe’ and the LOLA core database as ‘regions database’^48^. The activeDHS_universe data includes a universe set of all active REMs as defined by cross cell-type DNase hypersensitivity experiments and the LOLA core database incorporates datasets from public sources such as ENCODE, Cistrome, and CODEX.

### Motif enrichment analysis

Motif analyses around peak regions for the intersection of MYB peaks between three biological replicates (n = 22,780) as well as known motif enrichment analysis for the genomic coordinates found in the different C-groups was made using the HOMER program findMotifsGenome.pl (version 4.9)^82^ with the parameter ‘–size given’.

### Public ChIP-seq data from K562

Public K562 ChIP-seq data for various TFs, co-factors and proteins of genomic relevance that were mapped to hg19 were obtained from the ENCODE project^33^. We considered those dataset that have good quality as indicated by the various quality measures reported in the ENCODE database. The list of dataset with their ENCODE ID are listed in supplementary information (Supplementary Table S1).

### TF co-localization analyses

TF co-occupancy was investigated, by generating pairwise intersection profiles between MYB peaks in K562 and publicly available ChIP-seq peaks for other targets in K562 using the intervene pairwise module with the parameters ‘intervene pairwise –compute jaccard’. The pairwise intersection heatmap was generated from the resulting Jaccard similarity matrix using hierarchical clustering of both rows and columns using complete method and Euclidean distance with the intervene Shiny App^78^.

TF co-localization was investigated using the Gsuite HyperBrowser^51^. First the MYB ChIP-seq peaks overlapping from three replicates were converted into Gsuite format from bed formatted files using the Gsuite HyperBrowser conversion tool. Next, publicly available K562 ChIP-seq peaks from various TFs (Supplementary Table S1) in bed format were uploaded to the HyperBrowser and a Gsuite collection was generated from these public datasets. Genomic co-localization was investigated using tetrachoric correlation of a query track base-pairs (the public ChIP-seq data) against a reference track base-pairs (the MYB peaks). Track position randomization across the genome was performed by employing Monte Carlo (MC) simulations while preserving elements of the reference track and inter-element distances within the query tracks. Moderate resolution of global and local p-values were kept for MC false discovery rate (MCFDR) sampling depth. The reported tetrachoric coefficient for pairs of ChIP-seq data was normalized to the length of the overlapping genomic intervals in order to account for the variances of segments of intervals used for computing the pair-wise similarities. Regions co-occupied by MYB and that of the top five co-localizing TFs were identified using bedtools intersect and the resulting genomic coordinates were annotated using STITCHIT^47^. STITCHIT identified gene lists for these co-occupied regions are then used to investigate GO-term enrichment using PANTHER v11.

### Identification of MYB responsive “pioneer” and “non-pioneer” genes

We took the differential expression table from our recently published transcriptome data of K562 cells under different knockdown (KD) and rescue conditions of MYB (GEO accession: GSE85187) from^30^. We used the list of all genes that showed significant differential expression upon KD of MYB as our reference to differentiate between MYB responsive “pioneer” and “non-pioneer” genes. Here we defined the “pioneer genes” as those genes whose expression profile is affected upon KD of endogenous MYB expression and are rescued by ectopic expression of the wild type MYB (WT hcM) but not by the pioneering function deficient D152V mutant of MYB (D152V hcM). In contrast, we defined the MYB-responsive “non-pioneer” genes as those genes whose expression profile is affected by KD of endogenous MYB but this MYB KD related phenotype is rescued by ectopic expression of both the WT MYB and D152V. Heatmap showing the expression profiles of the MYB responsive pioneer genes were generated using the ClustVis webtool^83^. Each row in the cluster heatmaps represents a gene where rows were centred and unit variance scaling was applied to rows. Both rows and columns were clustered using correlation distance and average linkage. List of STITCHIT annotated MYB peaks were used to investigate overlaps between MYB-responsive “pioneer” genes and MYB-responsive “non-pioneer” genes.

## Supplementary Information

Supplementary Information

## Data Availability

The datasets generated and analysed during the current study are available in the GEO repository [accession number: GSE124541]. The ChIP-seq processing was performed in an automated workflow using snakemake version (version 5.5.4)^75^. The snakemake code used for the ChIP-seq processing can be found in the following GitHub repository (https://github.com/rblemma/K562_Myb_ChIP-seq).
