# Supplementary material for: Chromatin occupancy and target genes of the haematopoietic master transcription factor MYB
Source: Sci Rep. 2021 Apr 26;11:9008. doi: 10.1038/s41598-021-88516-w (PMC8076236; doi:10.1038/s41598-021-88516-w)
Supplement: Supplementary file 1 — Supplementary Information [file 41598_2021_88516_MOESM1_ESM.pdf]

Supplementary Information

Chromatin occupancy and target genes of the haematopoietic master transcription factor MYB

Roza B. Lemma[#,1,2], Marit Ledsaak[#,1,3], Bettina M. Fuglerud[1,4,5], Geir Kjetil Sandve[6], Ragnhild Eskeland[1,3,7] and Odd S. Gabrielsen[1,*]

[1]Department of Biosciences, University of Oslo, PO Box 1066 Blindern, 0316 Oslo, Norway.
[2]Centre for Molecular Medicine Norway (NCMM), Nordic EMBL Partnership, University of Oslo, 0318 Oslo, Norway.
[3] Institute of Basic Medical Sciences, Department of Molecular Medicine, University of Oslo, PO Box 1112 Blindern, 0317 Oslo, Norway.
[4]Terry Fox Laboratory, BC Cancer, Vancouver, BC V5Z 1L3, Canada.
[5]Department of Medical Genetics, University of British Columbia, Vancouver, BC V6T 1Z4, Canada.
[6]Department of Informatics, PO Box 1080 Blindern, University of Oslo, Oslo, 0371 Norway.
[7]Centre for Cancer Cell Reprogramming, Institute of Clinical Medicine, Faculty of Medicine, University of Oslo, Oslo, Norway.

[#] These two authors contributed equally to the work.

[*]Corresponding author
Email: o.s.gabrielsen@ibv.uio.no
Phone: +47 22 85 73 46 (office), +47 415 60 130 (mobile)

Supplementary Figure S1

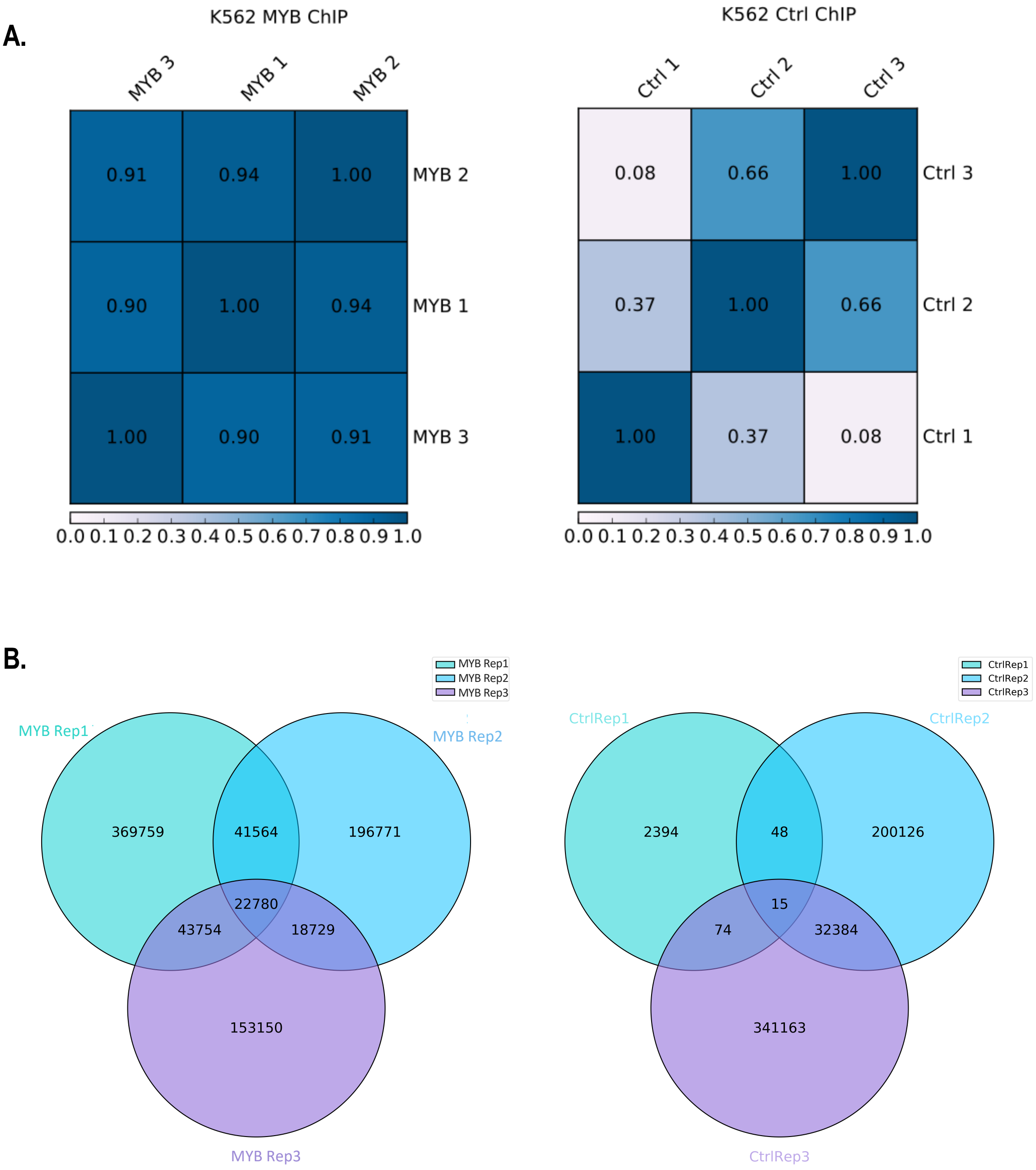

Supplementary Figure S1. Correlation of MYB ChIP-seq peaks in K562 from three biological replicates. A. Correlation heatmaps showing Pearson correlation of ChIP-seq peaks between three biological replicates from both control K562 cell line (right panel) and K562 cell line stably expressing N-terminally 3×Ty1-tagged full length MYB (left panel). The heatmaps were generated using the plotCorrelation program in deepTools2 v3.3.0 [1]. **B.** Overlap of ChIP-seq derived peaks between three biological replicates obtained from both control K562 cell line (right panel) and K562 cell line stably expressing the N-terminally 3×Ty1-tagged full length MYB (left panel). We used Intervene v0.6.4 [2] to investigate at least 50% physical overlap between the three biological replicates in both control and MYB ChIP-seq data.

Supplementary Figure S2

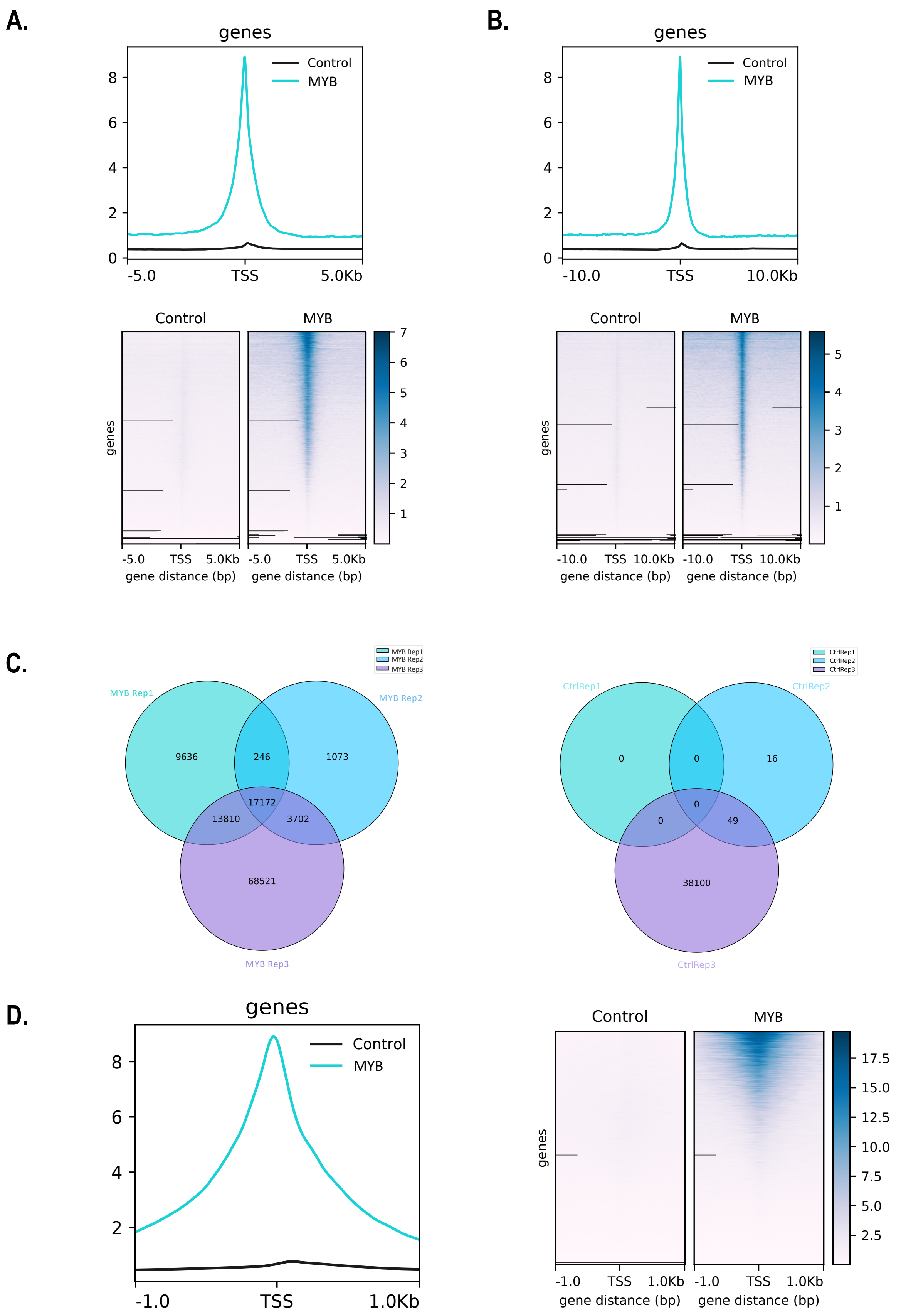

Supplementary Figure S2. Comparison of average ChIP-seq signals in K562 cells from the control cell line versus the stable cell line expressing N-terminally 3×Ty1-tagged full length MYB. A. A line plot indicating the intensity of MYB ChIP-seq signals at and ± 5 kb around the TSS of all genes. The heatmap below shows aggregated ChIP-seq signals at and ± 5 kb around the TSS of all genes. **B.** A line plot indicating the intensity of MYB ChIP-seq signals at and ± 10 kb around the TSS of all genes. The heatmap below shows aggregated ChIP-seq signals at and ± 10 kb around the TSS of all genes. We used Ensembl human reference genome annotation (GRCh37 release 87) as regions for calculating the ChIP-seq signal enrichment at and ± 5 kb as well as ± 10 kb around the TSSs of all genes. **C.** Overlap of ChIP-seq derived peaks with q-value cut-off < 0.005 between three biological replicates obtained from both control K562 cell line (right panel) and K562 cell line stably expressing the N-terminally 3×Ty1-tagged full length MYB (left panel). We used Intervene v0.6.4 [2] to investigate at least 50% physical overlap between the three biological replicates in both control and MYB ChIP-seq data. This additional ChIP-seq peak calling step was included to demonstrate that the present ChIP-seq data are of very high quality. Even at an extremely stringent cut-off involving a very low q-value in combination with considering only peaks that are consistently present in all three individual biological replicates, we report a large number of high quality MYB ChIP-seq peaks. **D.** Line plot and aggregated heatmap for the same MYB ChIP-seq signals as shown in panel C (Left panel) above. The line plot (right panel) indicates the intensity of the MYB ChIP-seq signals at and ±1 kb around the TSS of all genes using the same data. The heatmap shows aggregated ChIP-seq signals at and ± 1 kb around the TSS of all genes. The line plots and heatmaps were generated using deepTools2 v3.3.0 [1].

Supplementary Figure S3

LMO2 with upstream enhancer

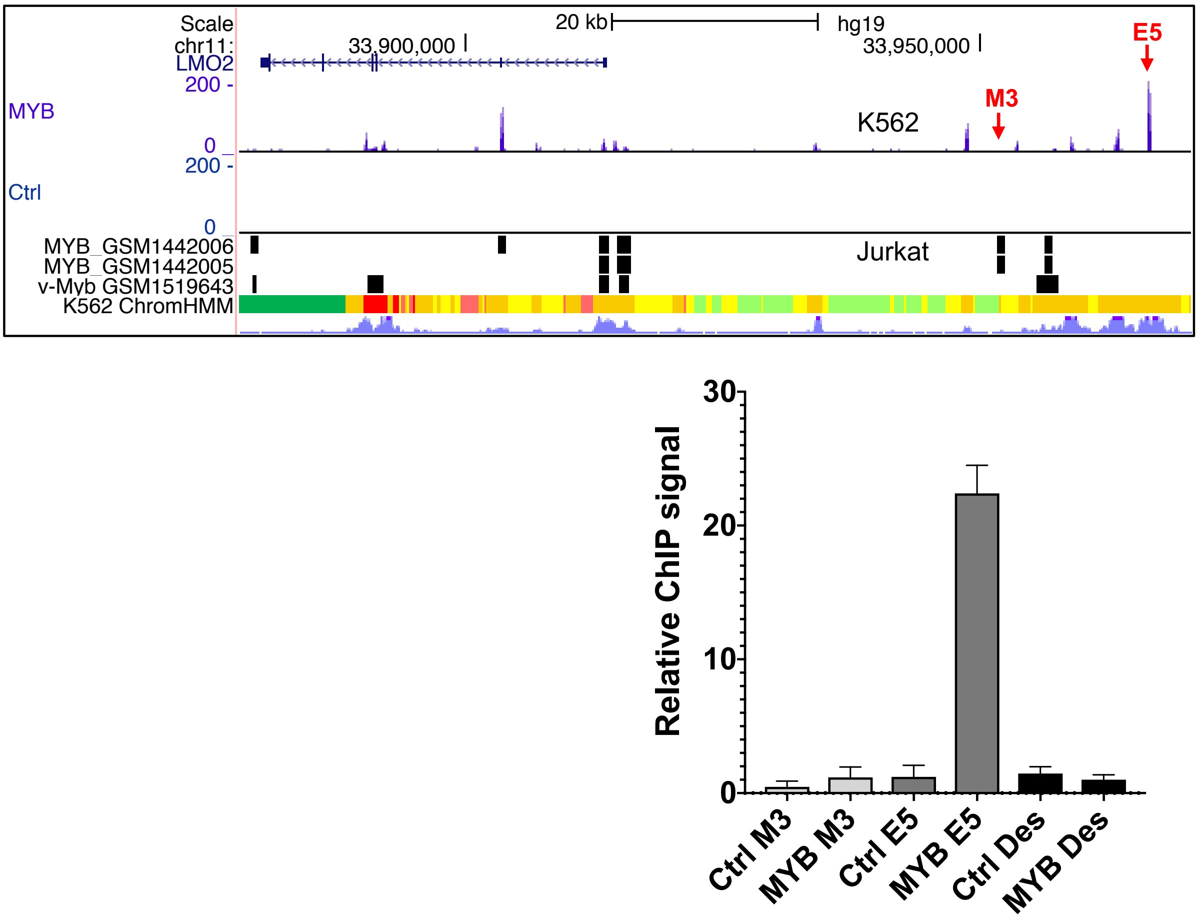

Supplementary Figure S3. Validation of MYB occupancy at the *LMO2* enhancer using ChIP-qPCR. As an independent validation assay, selected peaks identified in ChIP-seq were tested by ChIP-qPCR. Chromatin immunoprecipitations were performed with K562 cells stably expressing pEFneo-3×Ty1-MYB (marked MYB) and control pooled cell lines with stable expression of pEF1neo-3×Ty1 (marked Ctrl) as described in the Methods section. Occupancies were analysed by amplifying two specific regions (designated M3 and E5) from the *LMO2* enhancer by quantitative real-time PCR. M3 is a region with a ChIP-signal in Jurkat cells (track GSM1442005 with MYB clone 1-1 antibody Millipore 05-175, track GSM1442006 with MYB antibody Abcam AB45150) [3], but not in K562 cells according to the present ChIP-seq data. The opposite is the case for the E5 region, as illustrated in the upper panel. An unrelated DNA region was used as negative control, a region from a gene desert region (marked Des) [4]. The results are calculated from three biological replicates (each measured with three technical replicates) and are expressed in relative units setting the occupancy of MYB at the gene desert region to 1. The occupancies are given as mean with SEM (lower panel).

Supplementary Figure S4

C1

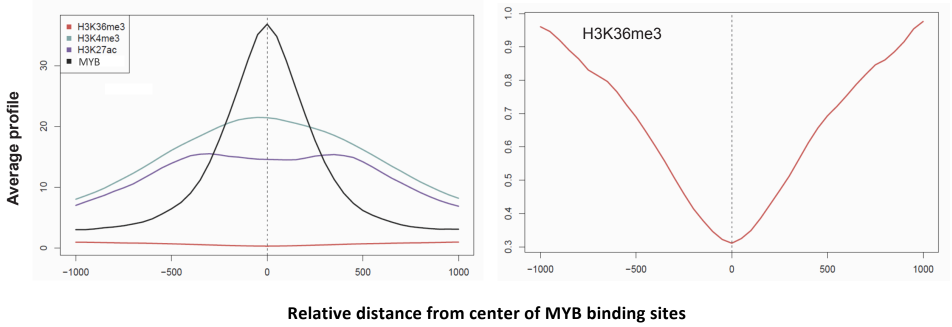

C2

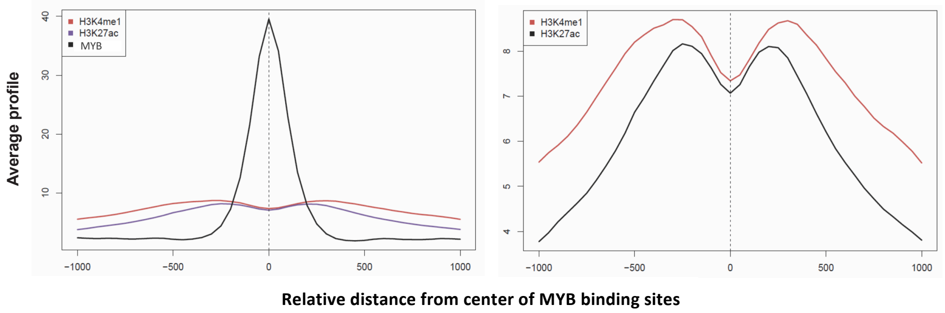

MYB signal in all clusters

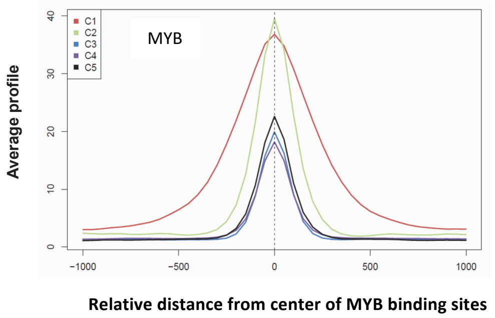

Supplementary Figure S4. MYB ChIP-seq signals along with signals from K562 histone marks aggregated at the center of MYB binding sites. Top panel: Average profile plots of H3K36me3, H3K4me3, and H3K27ac ChIP-seq signals within a 2 kb window around the summit of MYB bound regions in cluster C1 (left). The H3K36me3 signals are highlighted in the right panel (note the difference in y-axis scale). Middle panel: Average profile plots of H3K4me1 and H3K27ac ChIP-seq signals within a 2 kb window around the summit of MYB bound regions in cluster C2 (left). H3K4me1 and H3K27ac signals without MYB ChIP-seq signals are highlighted in the right panel. Bottom panel: Average profile plot of MYB ChIP-seq signals within a 2 kb window around the summits of MYB bound regions in each cluster C1-C5 from Fig. 3.

Supplementary Figure S5

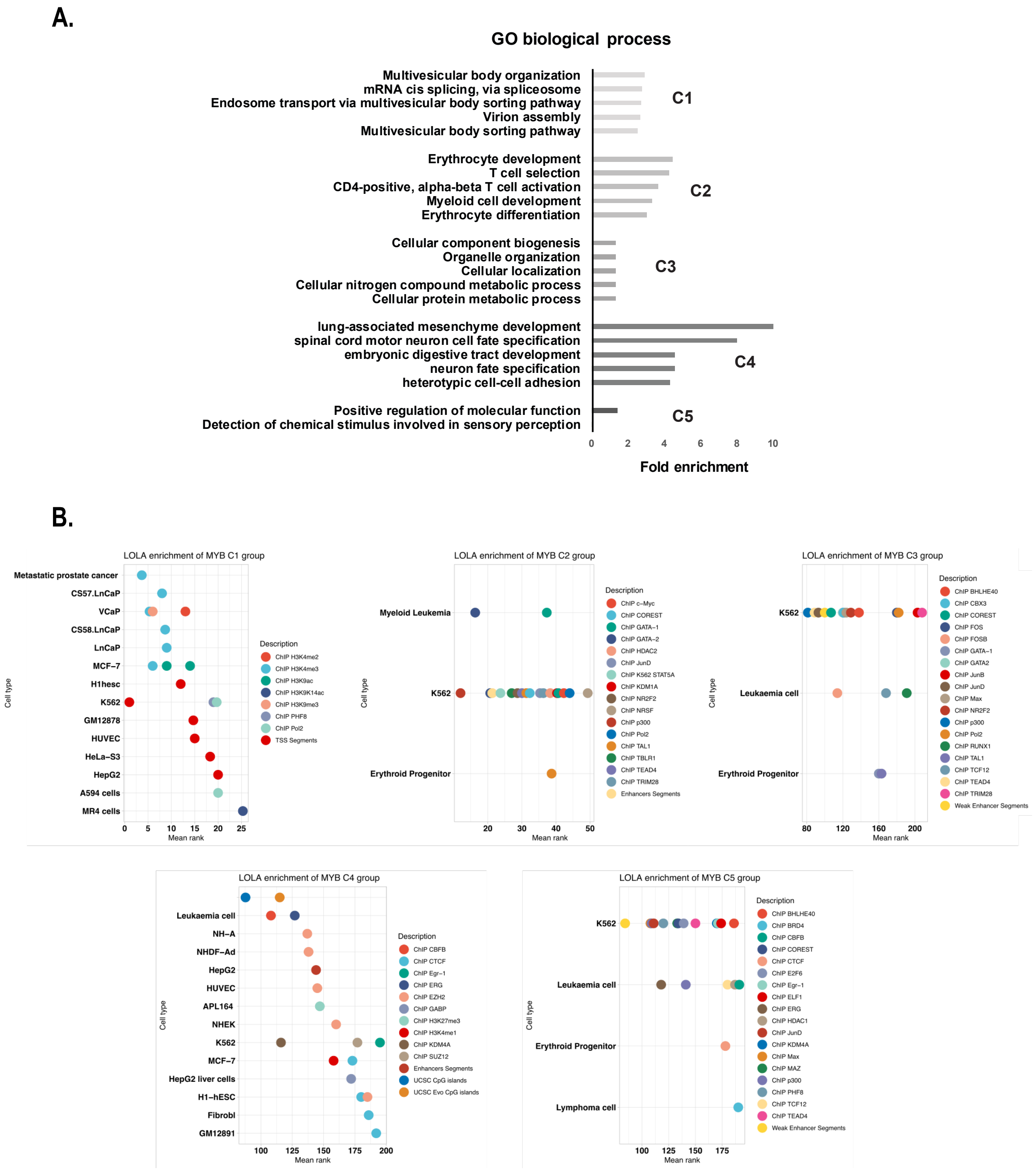

Supplementary Figure S5. **Enrichment analysis of the C-groups**. **A.** Top five enriched (or the only enriched for C5) biological processes for genes associated with MYB bound regions in each cluster C1-C5. GO-term enrichment was analysed using PANTHER v11 [5]. **B.** Enrichment of MYB occupied regions for the individual C-groups. We employed the LOLAweb regions enrichment analysis tool to compare the present genomic MYB profiles with other epigenetic profiles in the public domain. In each C-group, the enrichment of MYB occupied regions to a set of regions from publicly available genomic datasets accessed through the LOLAcore database, are ranked according to their similarity score [6].

Supplementary Figure S6

A.

Motif for cluster C3	Name	P-value	% of target seq with motif	% of background seq with motif
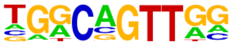	A-MYB	$1e^{-511}$	49.19%	15.45%
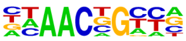	B-MYB	$1e^{-486}$	45.11%	13.46%
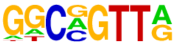	MYB	$1e^{-476}$	50.33%	17.06%
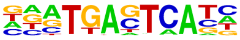	Fra1	$1e^{-109}$	13.68%	4.45%
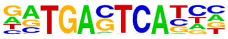	c-Jun	$1e^{-107}$	9.54%	2.32%
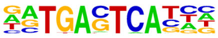	ATF3	$1e^{-106}$	15.43%	5.54%
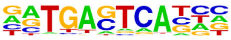	FOSL2	$1e^{-104}$	11.13%	3.15%
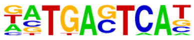	BATF	$1e^{-102}$	14.97%	5.36%
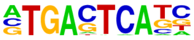	PU.1	$1e^{-67}$	16.99%	6.68%
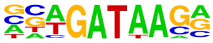	GATA4	$1e^{-60}$	13.78%	5.99%

B.

Motif for cluster C4	Name	P-value	% of target seq with motif	% of background seq with motif
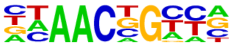	B-MYB	$1e^{-776}$	47.95%	13.64%
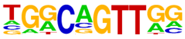	A-MYB	$1e^{-765}$	51.50%	16.04%
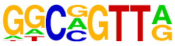	MYB	$1e^{-726}$	53.44%	18.02%
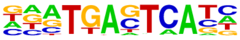	Fra1	$1e^{-191}$	14.70%	4.28%
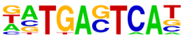	BATF	$1e^{-181}$	15.71%	5.02%
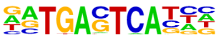	ATF3	$1e^{-179}$	16.03%	5.23%
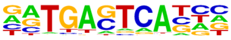	FOSL2	$1e^{-169}$	11.68%	3.09%
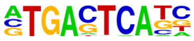	PU.1	$1e^{-164}$	17.23%	6.26%
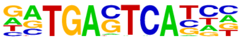	C-Jun	$1e^{-94}$	9.74%	2.23%
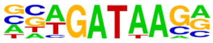	GATA4	$1e^{-85}$	13.29%	5.63%

Supplementary Figure S6. Enrichments of known motifs at MYB occupied regions in cluster C3 and C4. Enrichments of known motifs at MYB occupied regions in cluster C3 (panel A) and cluster C4 (Panel B) from Fig. 3A. Enriched binding motifs are arranged in descending order based on their p-value. Motif analyses around MYB binding sites in the C-groups were made using the HOMER program [7].

Supplementary Figure S7

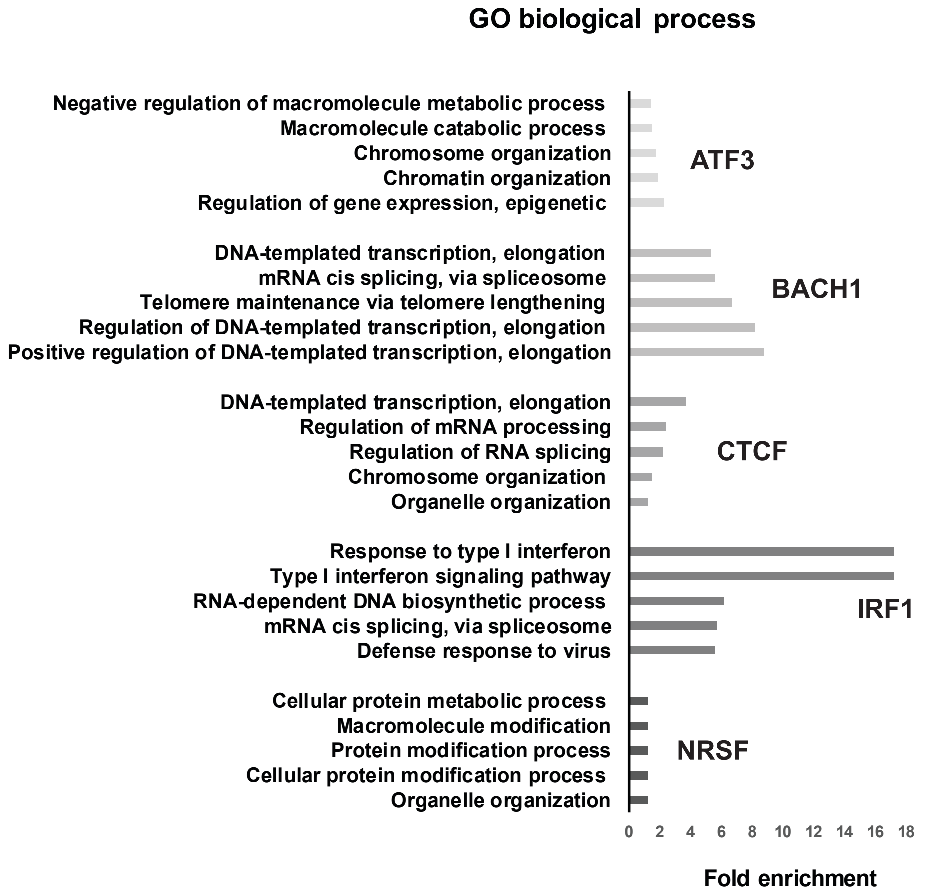

Supplementary Figure S7. Top enriched biological processes for genes associated with regions co-occupied by MYB and the top five co-localizing TF. Regions co-occupied by MYB and each of the top five co-localizing TFs shown in Fig.6 were identified by employing bedtools intersect (version 2.17.0) [8] on the corresponding pairs of the ChIP-seq peaks. The co-occupied regions were then associated with target genes using STITCHIT [9]. The STITCHIT associated genes were used to perform the GO-term analysis using PANTHER v11 [5].

Supplementary Figure S8

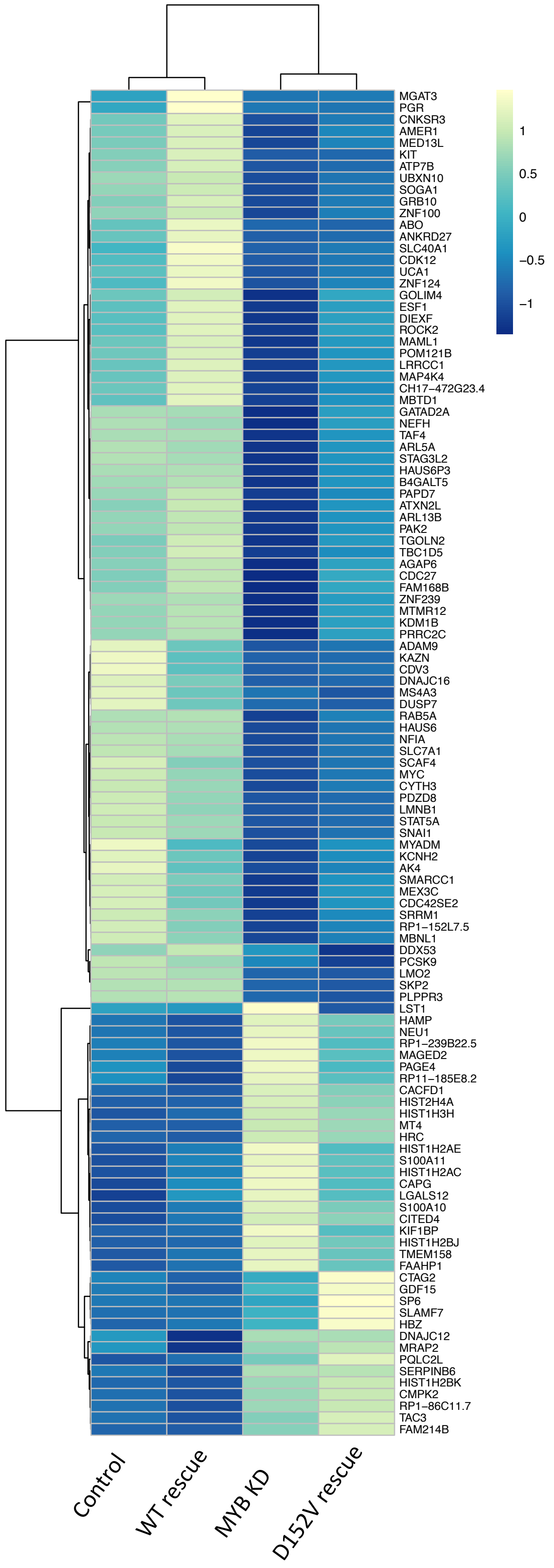

Supplementary Figure S8. Expression profiles of MYB responsive pioneer target genes. Hierarchical clustering of pioneer target genes of MYB (n=115) identified from the previously reported K562 RNA-seq data from our lab (GEO accession: GSE85187). The expressions of these pioneer target genes are affected in the endogenous MYB knockdown (KD) set and rescued by ectopic expression of wild-type (WT) MYB, whereas the pioneer function-deficient mutant, D152V MYB is unable to rescue the expression patterns. The cluster heatmap was generated using the ClustVis web tool [10]. Each row represents a single pioneer target gene [11].

Supplementary Figure S9

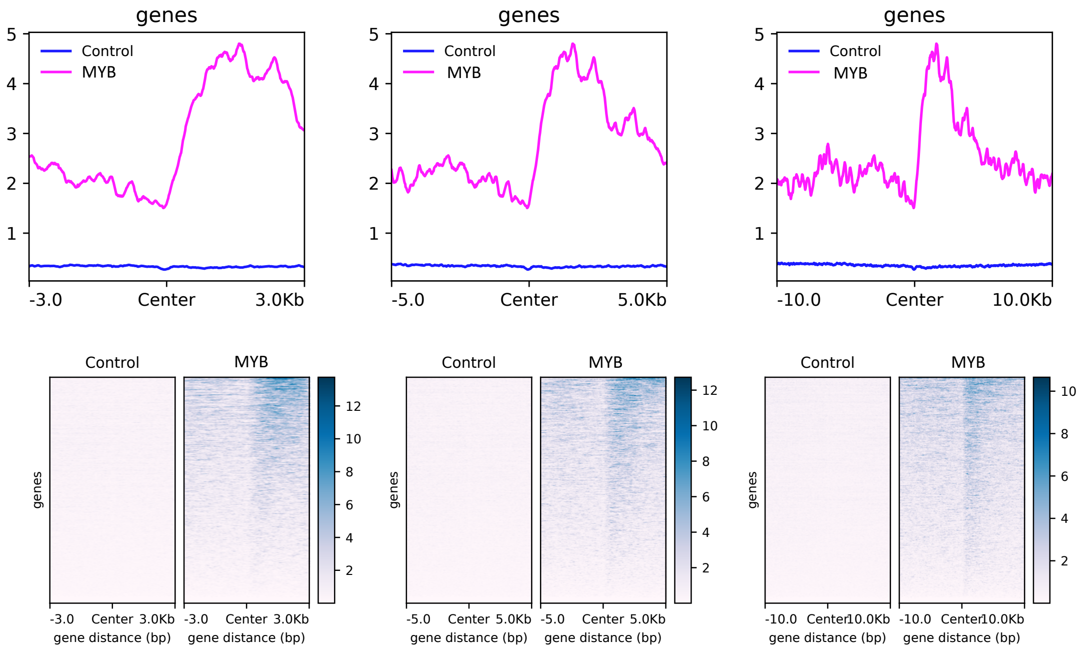

Supplementary Figure S9. MYB ChIP-seq signals around the K562 super enhancer (SE) elements. The MYB ChIP-seq signals at and ± 3 kb, 5 kb and 10 kb around the centre of K562 SE elements reported by Qian et al. [12] are shown. The line plots indicate the intensity of the MYB ChIP-seq signals at and surrounding the indicated genomic positions. The heatmaps show the ChIP-seq signals in the indicated genomic positions. The line plots and heatmaps were generated using deepTools2 v3.3.0 [1].

Supplementary Figure S10

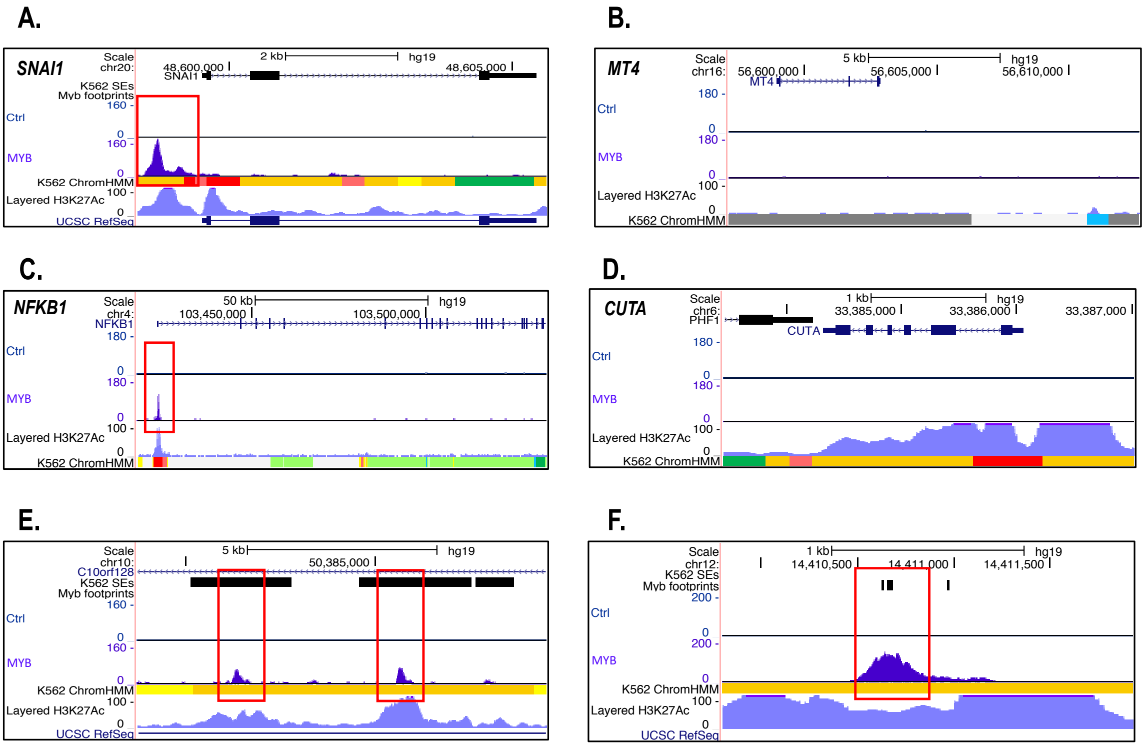

Supplementary Figure S10. Example tracks corresponding to the overlapping and non-overlapping regions reported on Fig.7. A. MYB occupancy at the promoter of the *SNAI2* locus illustrating an example of a direct pioneer target gene of MYB taken from the set reported in Fig. 7A (n=102). **B.** An example of an indirect pioneer target gene of MYB, *MT4*, taken from the set reported in Fig. 7A (n=13). A UCSC track at this locus illustrates the absence of MYB occupancy at this region. **C.** An example of direct non-pioneer target gene of MYB, *NFKB1*, taken from the set reported in Fig.7B (n=423). A UCSC track shows the occupancy of MYB at the TSS of *NFKB1*. **D.** An example of indirect non-pioneer target gene of MYB, *CUTA*, taken from the set reported in Fig.7B (n=430). A UCSC track at this locus illustrates the absence of MYB occupancy at this region. **E.** An example of the overlap between MYB occupied regions and Super-enhancer elements taken from the list in Fig.7D. **F.** An example of the overlap between MYB occupied regions and MYB footprint taken from the set reported in Fig.7C. Visualization of the tracks were made using the UCSC genome browser [13].

Supplementary Figure S11

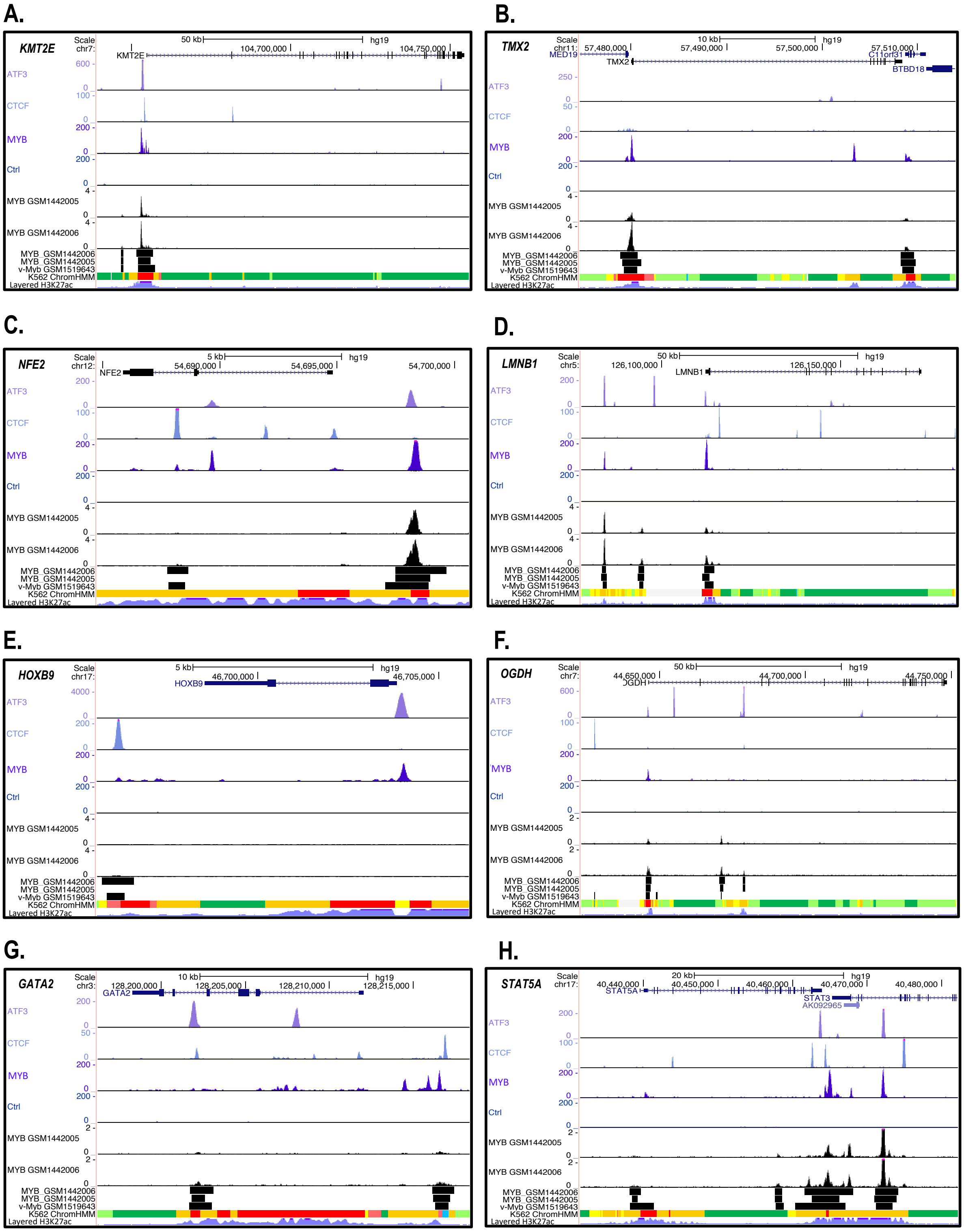

Supplementary Figure S11. Example tracks highlighting co-occupancy of MYB with the top two co-localizing factors (ATF3 and CTCF). In addition, publicly available ChIP-seq tracks for MYB in Jurkat cells (track GSM1442005 with MYB clone 1-1 antibody Millipore 05-175, track GSM1442006 with MYB antibody Abcam AB45150) and in MOLT3 cells (track GSM1519643 with MYB antibody Abcam ab45150) are added for some of the MYB target genes reported in Fig.1C, Fig.4C and Fig.8. **A.** MYB, ATF3, CTCF, Jurkat MYB and MOLT3 MYB ChIP-seq peak co-occupancy at the *KMT2E* locus **B.** MYB, CTCF, Jurkat MYB and MOLT3 MYB ChIP-seq peak co-occupancy at the *TMX2* locus. **C.** MYB, ATF3,

Jurkat MYB and MOLT3 MYB ChIP-seq peak co-occupancy at the *NFE2* locus. **D.** MYB, ATF3, Jurkat MYB and MOLT3 MYB ChIP-seq peak co-occupancy at the *LMNB1* locus **E.** MYB, ATF3 and CTCF co-occupancy at *HOXB9* locus. **F.** MYB, ATF3, Jurkat MYB and MOLT3 MYB ChIP-seq peak co-occupancy at the *OGDH* locus. **G.** MYB, CTCF, Jurkat MYB and MOLT3 MYB ChIP-seq peak co-occupancy at the *GATA2* locus. **H.** MYB, CTCF, Jurkat MYB and MOLT3 MYB ChIP-seq peak co-occupancy at the *STAT5A* locus. The Jurkat MYB and MOLT3 MYB ChIP-seq data are obtained from Mansour et al. [3]. The GEO accessions for ChIP-seq data obtained from Mansour et al. are displayed as track names on the UCSC genome browser. Visualization of the tracks were made using the UCSC genome browser [13].

Supplementary table S1. K562 publicly available ChIP-seq data collected from ENCODE [14, 15] used in the current study.

Public K562 ChIP-seq data	ENCODE-DCC accessions
E2F1	ENCFF998YJY
CTCFL	ENCFF962FQM
CTCF	ENCFF738TKN
BACH1	ENCFF319BCP
POLR2A	ENCFF947KPB
RCOR1	ENCFF484CKD
ZMIZ1	ENCFF597DIY
HCFC1	ENCFF474NLG
SIX5	wgEncodeAwgTfbsHaibK562Six5Pcr1xUniPk
TBP	ENCFF380FJL
TAF7	wgEncodeAwgTfbsHaibK562Taf7sc101167V0416101UniPk
GATA1	wgEncodeAwgTfbsSydhK562Gata1UcdUniPk
STAT2	wgEncodeAwgTfbsSydhK562Stat2Ifna30UniPk
THAP1	wgEncodeAwgTfbsHaibK562Thap1sc98174V0416101UniPk
FOSL1	wgEncodeAwgTfbsHaibK562Fosl1sc183V0416101UniPk
c-Fos	wgEncodeSydhTfbsK562CfosStdPk
HDAC2	wgEncodeAwgTfbsHaibK562Gata2sc267Pcr1xUniPk
SETDB1	wgEncodeSydhTfbsK562Setdb1UcdPk
BCLAF1	wgEncodeAwgTfbsHaibK562Bclaf101388Pcr1xUniPk
NFYA	wgEncodeSydhTfbsK562NfyaStdPk
HMGN3	ENCFF002CWT
c-Jun	cwgEncodeSydhTfbsK562CjunStdPk
SP1	ENCFF300XUA
NRF1	ENCFF992QZV
STAT5A	ENCFF696KPD
NRSF(REST)	ENCFF895QLA
ATF1	ENCFF030HWZ
GATA2	wgEncodeAwgTfbsHaibK562Gata2sc267Pcr1xUniPk
TBLR1	wgEncodeAwgTfbsSydhK562Tblr1nb600270IggrabUniPk
ATF3	ENCFF958KNK
CHD1	wgEncodeAwgTfbsBroadK562Chd1a301218aUniPk
CCNT2	ENCFF002CVU
TRIM28	ENCFF164HLQ
Sin3Ak-20	wgEncodeAwgTfbsHaibK562Sin3ak20V0416101UniPk
NR2F2	ENCFF255EOB
ETS1	ENCFF076YZO

SAP30	wgEncodeAwgTfbsBroadK562Sap3039731UniPk
GABP	wgEncodeAwgTfbsHaibK562GabpV0416101UniPk
GTF2B	ENCFF002CWR
CBX3	ENCFF455TDM
p300	ENCFF549TYR
YY1	ENCFF557DSM
UBTF	ENCFF671DOL
ELF1	ENCFF526EEI
HDAC1	ENCFF996CUX
EGR1	ENCFF004WYV
ELK1	ENCFF019PEL
TAF1-	ENCFF822FWM
ZBTB7A	ENCFF706ISJ
PML	ENCFF881QBT
JunD	ENCFF337DKJ
PLU1	ENCFF368TYM
ZC3H11A	ENCFF522JRK
MAX	ENCFF422NGZ
RBBP5	ENCFF379MPS
GTF2F1	ENCFF988FFD
IRF1	ENCFF388AJH
MAZ	wgEncodeAwgTfbsSydhK562Mazab85725IggrabUniPk
MXI1	ENCFF970LCB
PHF8	ENCFF626KTJ
CHD2	wgEncodeSydhTfbsK562Chd2ab68301IggrabPk
E2F6	GSM935597
TBLXR1	ENCFF048OBR
SPI1	ENCFF002CMJ
NFE2	ENCFF495MHZ

References

1. Ramirez, F., D.P. Ryan, B. Gruning, V. Bhardwaj, F. Kilpert, A.S. Richter, S. Heyne, F. Dundar, and T. Manke, *deepTools2: a next generation web server for deep-sequencing data analysis.* Nucleic Acids Res, 2016. **44**(W1): p. W160-5.
2. Khan, A. and A. Mathelier, *Intervene: a tool for intersection and visualization of multiple gene or genomic region sets.* BMC Bioinformatics, 2017. **18**(1): p. 287.
3. Mansour, M.R., B.J. Abraham, L. Anders, A. Berezovskaya, A. Gutierrez, A.D. Durbin, J. Etchin, L. Lawton, S.E. Sallan, L.B. Silverman, M.L. Loh, S.P. Hunger, T. Sanda, R.A. Young, and A.T. Look, *Oncogene regulation. An oncogenic super-enhancer formed through somatic mutation of a noncoding intergenic element.* Science, 2014. **346**(6215): p. 1373-7.
4. Barski, A., S. Cuddapah, K. Cui, T.Y. Roh, D.E. Schones, Z. Wang, G. Wei, I. Chepelev, and K. Zhao, *High-resolution profiling of histone methylations in the human genome.* Cell, 2007. **129**(4): p. 823-37.
5. Mi, H., X. Huang, A. Muruganujan, H. Tang, C. Mills, D. Kang, and P.D. Thomas, *PANTHER version 11: expanded annotation data from Gene Ontology and Reactome pathways, and data analysis tool enhancements.* Nucleic Acids Res, 2017. **45**(D1): p. D183-D189.
6. Nagraj, V.P., N.E. Magee, and N.C. Sheffield, *LOLAweb: a containerized web server for interactive genomic locus overlap enrichment analysis.* Nucleic Acids Res, 2018. **46**(W1): p. W194-W199.
7. Heinz, S., C. Benner, N. Spann, E. Bertolino, Y.C. Lin, P. Laslo, J.X. Cheng, C. Murre, H. Singh, and C.K. Glass, *Simple combinations of lineage-determining transcription factors prime cis-regulatory elements required for macrophage and B cell identities.* Mol Cell, 2010. **38**(4): p. 576-89.
8. Quinlan, A.R. and I.M. Hall, *BEDTools: a flexible suite of utilities for comparing genomic features.* Bioinformatics, 2010. **26**(6): p. 841-2.
9. Schmidt, F., A. Marx, M. Hebel, M. Wegner, N. Baumgarten, M. Kaulich, J. Göke, J. Vreeken, and M.H. Schulz, *Integrative analysis of epigenetics data identifies gene-specific regulatory elements.* bioRxiv, 2019: p. 585125.
10. Metsalu, T. and J. Vilo, *ClustVis: a web tool for visualizing clustering of multivariate data using Principal Component Analysis and heatmap.* Nucleic Acids Res, 2015. **43**(W1): p. W566-70.
11. Fuglerud, B.M., R.B. Lemma, P. Wanichawan, A.Y.M. Sundaram, R. Eskeland, and O.S. Gabrielsen, *A c-Myb mutant causes deregulated differentiation due to impaired histone binding and abrogated pioneer factor function.* Nucleic Acids Res, 2017. **45**(13): p. 7681-7696.
12. Qian, F.C., X.C. Li, J.C. Guo, J.M. Zhao, Y.Y. Li, Z.D. Tang, L.W. Zhou, J. Zhang, X.F. Bai, Y. Jiang, Q. Pan, Q.Y. Wang, E.M. Li, C.Q. Li, L.Y. Xu, and D.C. Lin, *SEanalysis: a web tool for super-enhancer associated regulatory analysis.* Nucleic Acids Res, 2019. **47**(W1): p. W248-W255.
13. Kent, W.J., C.W. Sugnet, T.S. Furey, K.M. Roskin, T.H. Pringle, A.M. Zahler, and D. Haussler, *The human genome browser at UCSC.* Genome Res, 2002. **12**(6): p. 996-1006.
14. Consortium, E.P., *An integrated encyclopedia of DNA elements in the human genome.* Nature, 2012. **489**(7414): p. 57-74.

15. Davis, C.A., B.C. Hitz, C.A. Sloan, E.T. Chan, J.M. Davidson, I. Gabdank, J.A. Hilton, K. Jain, U.K. Baymuradov, A.K. Narayanan, K.C. Onate, K. Graham, S.R. Miyasato, T.R. Dreszer, J.S. Strattan, O. Jolanki, F.Y. Tanaka, and J.M. Cherry, *The Encyclopedia of DNA elements (ENCODE): data portal update.* Nucleic Acids Res, 2018. **46**(D1): p. D794-D801.
